# Structural and functional insights into the DNA damage-inducible protein 1 (Ddi1) from protozoa

**DOI:** 10.1016/j.crstbi.2022.05.003

**Published:** 2022-05-26

**Authors:** Killivalavan Asaithambi, Iman Biswas, Kaza Suguna

**Affiliations:** Molecular Biophysics Unit, Indian Institute of Science, Bangalore, 560012, India

**Keywords:** Ddi1, RVP, Crystal structure, Flap, Active site, Inhibitor, Ddi1, DNA damage-inducible protein 1, RVP, retroviral protease, UBL, ubiquitin-like, UBA, ubiquitin-associated, HDD, helical domain of Ddi1, BLI, biolayer interferometry, NMA, normal mode analysis

## Abstract

Ddi1 is a multidomain protein that belongs to the ubiquitin receptor family of proteins. The Ddi1 proteins contain a highly conserved retroviral protease (RVP)-like domain along with other domains. The severity of opportunistic infections, caused by parasitic protozoa in AIDS patients, was found to decline when HIV protease inhibitors were used in antiretroviral therapy. Parasite growth was shown to be suppressed by a few of the inhibitors targeting Ddi1 present in these parasites. In this study, the binding of HIV protease inhibitors to the RVP domain of Ddi1 from *Toxoplasma gondii* and *Cryptosporidium hominis*; and the binding of ubiquitin to the ubiquitin-associated domain of Ddi1 from these two parasites were established using Biolayer Interferometry. The crystal structures of the RVP domains of Ddi1 from *T. gondii* and *C. hominis* were determined; they form homodimers similar to those observed in HIV protease and the reported structures of the same domain from *Saccharomyces cerevisiae*, *Leishmania major* and humans. The native form of the domain showed an open dimeric structure and a normal mode analysis revealed that it can take up a closed conformation resulting from relative movements of the subunits. Based on the crystal structure of the RVP domain of Ddi1 from *L. major*, a seven residue peptide inhibitor was designed and it was shown to bind to the RVP domain of Ddi1 from *L. major* by Biolayer Interferometry. This peptide was modified using computational methods and was shown to have a better affinity than the initial peptide.

## Highlights

• Crystal structures of the retroviral protease (RVP)-like domains of DNA damage inducible protein 1 (Ddi1) from *Toxoplasma gondii* and *Cryptosporidium hominis* have been determined.
• *Toxo*Ddi1-RVP and *Cryp*Ddi1-RVP crystal structures illustrate the conserved structural features with HIV protease.
• HIV protease inhibitors were found to bind to *Toxo*Ddi1-RVP and *Cryp*Ddi1-RVP in solution showing the potential to be developed as drugs for protozoal diseases.

## Graphical abstract

**Figure:**
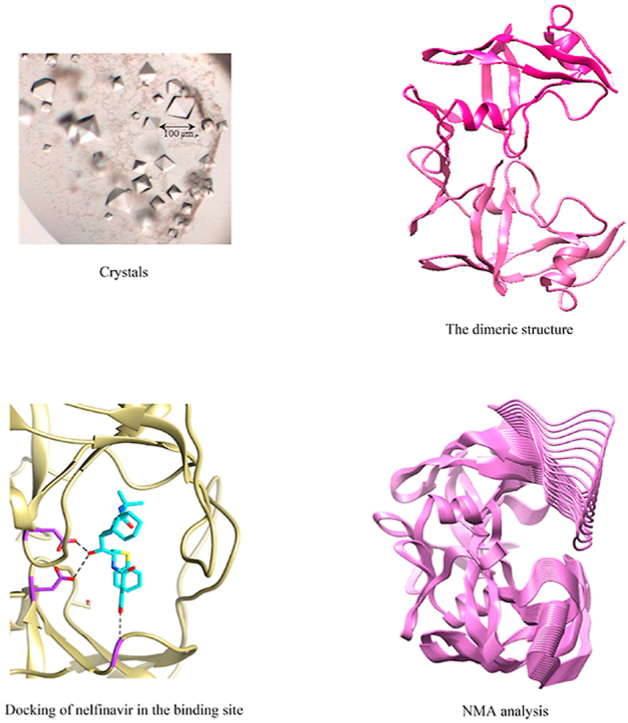

## 1 Introduction

The DNA damage-inducible protein 1 (Ddi1) is a multidomain protein that was originally identified as the product of a gene which expressed both MAG1 (3-methyladenine DNA glycosylase) and Ddi1 under the control of a bidirectional promoter, in *S. cerevisiae* or yeast (Liu and Xiao, 1997). This protein belongs to a family of proteins known as ubiquitin receptors of the proteasome-mediated degradation pathway and take part in several cellular processes. Ddi1 degrades HO endonuclease and the F-box protein, Ufo1, to enable the regulation of cell cycle progression (Kaplun et al., 2006; Ivantsiv et al., 2006). By interacting with the t-SNARE and v-SNARE proteins, Ddi1 acts as a negative regulator of exocytosis (Lustgarten and Gerst, 1999; Marash and Gerst, 2003; Gabriely et al., 2008). In budding yeast, Ddi1 participates in S-phase checkpoint control by suppressing a temperature sensitive mutant of pds1 (Clarke et al., 2001). The transcription factors SKN-1A and Nrf1 (a homologue of SKN-1) were reported to be the natural substrates of Ddi1 in *Caenorhabditis elegans* (Lehrbach and Ruvkun, 2016) and human Ddi2 (a homologue of Ddi1) (Koizumi et al., 2016), respectively. Also, human Ddi1 and Ddi2 were found to maintain genome stability by removing RTF2 from replication forks (Kottemann et al., 2018). Ddi1 of yeast (yeastDdi1) was identified as a conserved metacaspase substrate (Bouvier et al., 2018). It has been found to assist in the removal of proteins from DNA-protein crosslinks, which hinder the replication process (Svoboda et al., 2019; Serbyn et al, 2020). YeastDdi1 was shown to cleave polyubiquitinated substrates, probably playing a role in compensating for the loss of proteasome function (Yip et al., 2020).

**Fig. 1:**
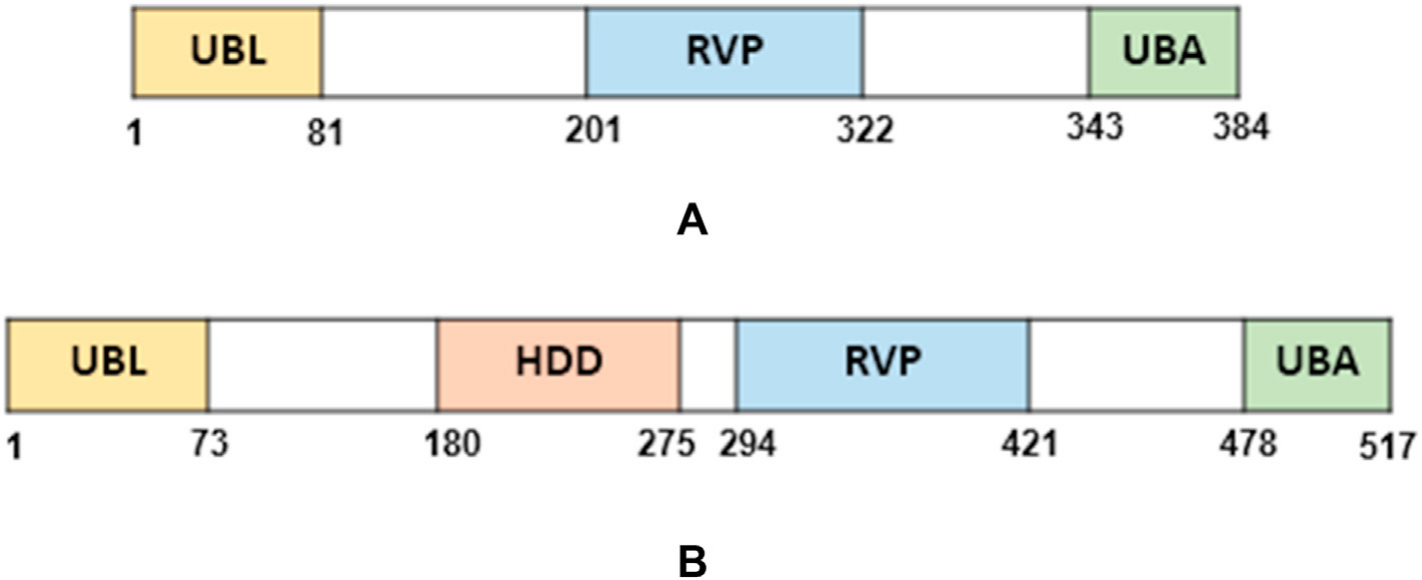
Domain organization of Ddi1 from A) *C. hominis* and B) *T. gondii*. UBL, ubiquitin-like domain; HDD, helical domain of Ddi1; RVP, retroviral protease-like domain; UBA, ubiquitin associated domain.

Ddi1 comprises three major domains: a ubiquitin-like (UBL) domain, a ubiquitin-associated (UBA) domain and a retroviral protease (RVP)-like domain; an additional domain preceding the RVP domain called the helical domain of Ddi1 (HDD), was identified in yeastDdi1 (Trempe et al., 2016) and humanDdi2 (Sivá et al., 2016). The domain organization of the proteins reported in the present study are shown in Fig. 1. Interestingly, the RVP domain is always present in Ddi1 but the UBA and/or UBL domain is absent in a few organisms (Nowicka et al., 2015; Krylov and Koonin, 2001; Sivá et al., 2016). The ubiquitin receptor family of proteins bind to ubiquitinated substrates through the UBA domain and deliver the substrates to the proteasome by interacting with the regulatory subunit of the 26S proteasome through the UBL domain. This facilitates the degradation of ubiquitinated proteins. The presence of the RVP domain makes Ddi1 unusual, as it is the only known protein among the ubiquitin receptor family of proteins to have this domain (Gabriely et al., 2008). A sequence comparison of Ddi1 from various organisms shows that the RVP domain is highly conserved. The NMR structures of the HDD domain revealed similarities to DNA binding domains from transcriptional regulators (Trempe et al., 2016; Sivá et al., 2016). Disorder prediction of Ddi1 revealed the presence of highly disordered regions between UBL and the HDD and between RVP and the UBA domains. This leads to high flexibility of the protein which may be important for the protein to mediate its functions associated with different domains (Aufderheide et al., 2015).

Interestingly, some of the AIDS patients treated by Highly Active Antiretroviral Therapy (HAART) acquired partial protection against leishmaniasis and other parasitic diseases. Later, this phenomenon was shown to be a consequence of the HIV protease inhibitors used in the therapy interacting with the Ddi1 present in parasites (Savoia et al., 2005; Valdivieso et al., 2007; Trudel et al., 2008; Li et al., 1998). It was reported that ritonavir and nelfinavir inhibit the growth of *T. gondii* which causes toxoplasmic encephalitis (Wang et al., 2019). The protease activity of Ddi1 of *T. gondii* was found to be important for virulence in mice (Zhang et al., 2020). This has provided a new direction to develop specific inhibitors to Ddi1 to kill the pathogen. In case of severe immunosuppression following HIV infection, the use of antiretroviral therapy caused restoration of mucosal immunity due to an increase in CD4 T-cells leading to cure of clinical cryptosporidiosis (Schmidt et al., 2001). Nelfinavir could be an effective drug to treat multiple myeloma and other cancers due to its ability to inhibit the activity of human Ddi2 (Gu et al., 2020; Fassmannova et al., 2020).

The first structure of the RVP domain of Ddi1 to be reported was from yeast (yeastDdi1-RVP) (PDB Code: 2I1A; Sirkis et al., 2006)*.* It was a homodimer, with each protomer having the retroviral protease fold; however, the loop region called the ‘flap’, which overhangs the active site could not be traced. In the second structure of the yeastDdi1-RVP (PDB Code: 4Z2Z; Trempe et al., 2016), the flap from one subunit of the dimer could be clearly defined as it interacts extensively with the N-terminal segment from an adjacent symmetry-related molecule, which mimics a substrate peptide and binds in the active site. In the structure of the RVP domain of human Ddi2 (humanDdi2-RVP) (PDB Code: 4RGH; Sivá et al., 2016), electron density could be seen for one of the flaps, which interacts with a symmetry-related molecule. In the structure of Ddi1-RVP from *L. major* (*Leish*Ddi1-RVP) (PDB Code: 5YQ8; Kumar and Suguna, 2018), the electron density corresponding to both the flaps was present; each flap of the dimer interacts with the active site residues of another dimer in the asymmetric unit via hydrogen bonds and salt bridges. Even though a few of the Ddi1-RVP flap regions could not be traced in these reported structures, some of them could be clearly defined when this region interacted with other molecules in the crystal.

Though HIV protease inhibitors have detrimental effects on parasitic protozoa, they are not potent enough to be used for treating standalone diseases unless their efficacy is substantially improved. More structural insights on protozoal Ddi1 in the apo and inhibitor-bound forms will aid in this process. With this in view, we have taken up structure analysis of the RVP domain of Ddi1 from two protozoa, *T. gondii* and *C. hominis*. In addition, we performed a normal mode analysis (NMA) on both the structures and molecular dynamics (MD) simulation studies on the structure of the Ddi1-RVP domain from *T. gondii* to investigate the dynamics of this domain with a focus on the flap region. We also carried out *in vitro* binding studies of Ddi1 with HIV protease inhibitors using biolayer interferometry (BLI) and molecular docking of HIV protease inhibitors to the structures of Ddi1-RVP from *T. gondii* and *C. hominis* to probe Ddi1-inhibitor interactions. In this paper, we report the crystal structures of Ddi1-RVP from *T. gondii* and *C. hominis*; and binding of HIV-1 PR inhibitors, nelfinavir and saquinavir to Ddi1-RVP from *T. gondii* and *C. hominis*, respectively. This will provide a starting point for the development of antiprotozoal drugs with Ddi1 as the target.

**Fig. 2:**
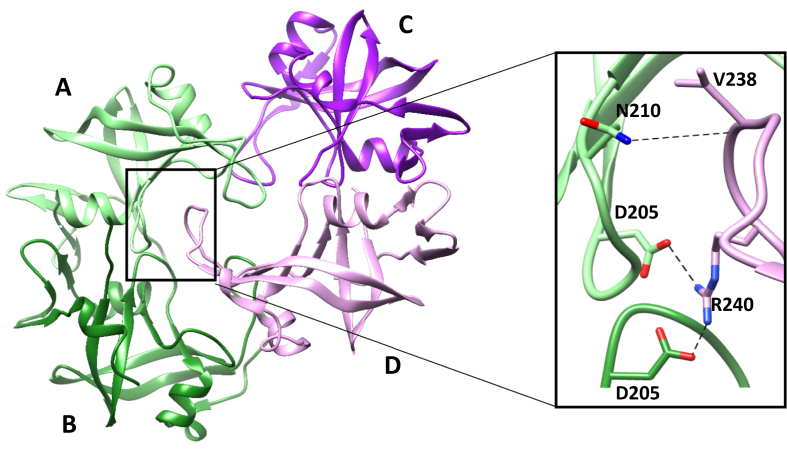
Interaction of the flap region with the active site of the neighboring dimer in the crystal structure of *Leish*Ddi1-RVP.

In the structure of the RVP domain of Ddi1 from *L. major* (Kumar and Suguna, 2018), it was observed that four polypeptide chains are arranged as two dimers in the asymmetric unit (Fig. 2) and the flap of one of the subunits in one dimer interacts with the active site of another dimer in the asymmetric unit via hydrogen bonds and salt bridges. Based on this observation, with an anticipation that a peptide comprising seven residues (GVGRQEI) of the flap region interacting with the other dimer may act as an inhibitor to the protein, we studied the binding of this heptapeptide to the RVP domain of Ddi1 from *L. major* and modified this peptide which showed better binding.

## 2 Materials and methods

### 2.1 Cloning, protein expression and purification

The Ddi1 genes from *T. gondii* (Gene ID: 7893859, 1581 bp) and *C. hominis* (Gene ID: 3413713, 1152 bp) were synthesized by GenScript, USA. From the full length *Toxo*Ddi1 gene, two constructs, *Toxo*Ddi1-RVP ​+ ​UBA (880–1551 bp) and *Toxo*Ddi1-RVP (880–1263 bp) were subcloned in pET-28a vector between *NheI* and *XhoI* sites with an N-terminal hexa-histidine tag. From the full length *Cryp*Ddi1 gene, two constructs, *Cryp*Ddi-RVP ​+ ​UBA (595–1137 bp) and *Cryp*Ddi-RVP (595–978 bp) were subcloned in pET-21b vector between *NheI* and *HindIII* sites with a C-terminal hexa-histidine tag. The full length Ddi1 construct, *Leish*Ddi1-fl (1–390 aa) was cloned in pET-28a vector between *NdeI* and *HindIII* sites with an N-terminal hexa-histidine tag (Kumar and Suguna, 2018). The plasmids containing the gene of interest were transformed into *E. coli* BL-21 (DE23) competent cells and plated on LB agar medium containing kanamycin. A single colony of the transformed *E. coli* BL-21 ​cells was inoculated in LB medium containing kanamycin and grown at 37 ​°C for 10–12 ​h. One percent of the overnight culture was inoculated in the LB medium and grown at 37 ​°C until the optical density at 600 ​nm reached ∼0.6. Protein expression was induced by adding 0.25 ​mM isopropyl β-D-1-thiogalactopyranoside (IPTG) and the cells were further grown at 18 ​°C for 16–20 ​h. The cells were harvested by centrifugation at 6000 ​rpm for 10 ​min. The pelleted cells were suspended in a lysis buffer containing 20 ​mM Tris/HCl (pH 7.5) and 300 ​mM NaCl. The cells lysed by sonication were centrifuged at 13000 ​rpm for 45 ​min. The supernatant was passed through a pre-equilibrated Ni-NTA column (GE Healthcare, Uppsala, Sweden), 20 column volumes of buffer containing 20 ​mM Tris/HCl (pH 7.5), 300 ​mM NaCl and 10 ​mM imidazole were passed through the column to remove impurities bound non-specifically to the column. The proteins were eluted with a buffer containing 20 ​mM Tris/HCl (pH 7.5), 300 ​mM NaCl and 300 ​mM imidazole. Further purification was carried out by gel filtration chromatography using a 16/60 Superdex 200 gel filtration column (GE Healthcare) equilibrated with a buffer containing 20 ​mM Tris/HCl (pH 7.5), 100 ​mM NaCl and 2 ​mM β-mercaptoethanol using a BioLogic DuoFlow FPLC system from Bio-Rad (Hercules, CA, USA). The purity of the proteins was examined by running 15% SDS-PAGE (Supplementary Fig. 1).

### 2.2 Activity assay and inhibition studies by fluorescence resonance energy transfer (FRET)

The activity assay was carried out using HIV protease substrate-1 (Sigma-aldrich). This is a synthetic peptide having the sequence Arg-Glu(EDANS)-Ser-Gln-Asn-Tyr-Pro-Ile-Val-Gln-Lys(DABCYL)-Arg. It contains the HIV protease cleavage site (Tyr-Pro) and two covalently modified amino acids for the detection of the proteolytic activity: the fluorophore EDANS was attached to the glutamic acid residue on the N-terminal side and the acceptor chromophore DABCYL to the lysine residue on the C-terminal side of the scissile bond. EDANS excited with light at 340 ​nm emits light at 490 ​nm which gets absorbed by the DABCYL group. Cleavage of the peptide separates the modified amino acids quenching FRET and emission could be detected at 490 ​nm *Toxo*Ddi1-RVP and *Cryp*Ddi1-RVP were added in different concentrations to 2 ​μM of HIV protease substrate-1 in a buffer containing 0.1M sodium acetate pH 4.5, 1.0 ​M sodium chloride, 1.0 ​mM EDTA, 1.0 ​mM DTT and 10% DMSO. To study the inhibition of proteolytic activity, 0.5 ​mM of HIV protease inhibitors, saquinavir, nelfinavir, amprenavir, darunavir, indinavir and ritonavir were added to the reaction mixture containing 20 ​μM of protein along with the substrate. A similar reaction mixture containing 5 ​μM of HIV protease tethered dimer instead of Ddi1-RVP was used as a positive control for the experiments. All the samples were incubated at 37 ​°C for 1 ​h and the fluorescence emission was recorded at 490 ​nm.

### 2.3 Bio-layer interferometry (BLI)

The binding of HIV protease inhibitors to *Toxo*Ddi1-RVP and *Cryp*Ddi1-RVP, binding of monoubiquitin and K48-linked diubiquitin to *Toxo*Ddi1-RVP ​+ ​UBA and *Cryp*Ddi1-RVP ​+ ​UBA, and binding of peptides to *Leish*Ddi1-fl were measured by BLI using an Octet Red96 system (Pall ForteBio, Fremont, CA, USA). To study the binding of HIV protease inhibitors, 10 ​μM of His-tagged protein was immobilized on the Ni-NTA sensor tip which was then dipped in a solution containing 100 ​mM MES buffer (pH 5.5), 150 ​mM NaCl, 0.5% BSA, 0.05% Tween 20 and inhibitors at 75 ​μM, 150 ​μM, 300 ​μM and 600 ​μM concentrations for 3 ​min at each concentration. To study the binding of monoubiquitin and K48-linked diubiquitin, 1 ​μM of monoubiquitin/K48-linked diubiquitin was immobilized on the AR2G sensor tip via amine coupling mechanism and dipped in protein of varying concentrations in 20 ​mM HEPES (pH 7.5) and 100 ​mM NaCl for 10 ​min at each concentration. To study the binding of peptides to *Leish*Ddi1-fl, 5 ​mM of peptide was immobilized on the AR2G sensor tip and dipped in protein of varying concentrations in 20 ​mM HEPES (pH 7.5) and 100 ​mM NaCl for 10 ​min at each concentration. Double referencing was performed in all the cases, to obtain more accurate results. A simple 1:1 Langmuir interaction model was used to fit the data.

### 2.4 High performance liquid chromatography (HPLC)

The enzymatic activities of *Toxo*Ddi1-RVP and *Cryp*Ddi1-RVP were also probed by HPLC (Agilent Technologies, Compact LC, 1200) equipped with single wavelength UV-detector at 490 ​nm and an Eclipse C18 analytical column ((150 ​mm ​× ​4.6 ​mm), particle size 3.5 ​μm)). Absorbance at a wavelength of 490 ​nm was recorded to detect the DABCYL group. The C-terminal fragment of the product should appear as a peak after cleavage. A reaction mixture consisting of 100 ​mM sodium acetate at pH 4.7, 1 ​M NaCl, 1 ​mM DTT and 22 ​μg/ml of *Toxo*Ddi1-RVP/*Cryp*Ddi1-RVP and 10 ​μM of HIV protease substrate-1 was incubated at 37 ​°C for 1 ​h and was analyzed by HPLC. For the detection of the chromophore, 0.1% of TFA (Trifluoro acetic acid) and acetonitrile at 50:50 ratio were used as a mobile phase with the flow rate of 0.8 ​ml/min. Absorbance at 490 ​nm was also recorded for 2 ​μM of the substrate alone, as a reference.

### 2.5 Kinetics assay

Enzyme kinetics assay was performed using a reaction mixture consisting of 100 ​mM sodium acetate buffer at pH 4.7, 1 ​M NaCl and 1 ​mM DTT and 22 ​μg/ml of *Toxo*Ddi1-RVP/*Cryp*Ddi1-RVP at increasing concentrations (2 ​μM, 4 ​μM, 6 ​μM, 8 ​μM, 10 ​μM, 20 ​μM, 40 ​μM, 60 ​μM, 80 ​μM and 100 ​μM) of HIV protease substrate-1. Cleavage of the HIV protease substrate-1 (Arg-Glu[EDANS]-Ser-Gln-Asn-Tyr-Pro-Ile-Val-Gln-Lys[DABCYL]-Arg) that occurs at Try-Pro was monitored. The reaction mixture was kept in a micro plate reader (Varioskan Flash; ThermoFisher Scientific) at 37 ​°C and the reading was taken every 60 ​s with 3 ​s stirring intervals for 60 ​min. The *λ*_excitation_ was 340 ​nm, and the *λ*_emission_ was 490 ​nm. A 96-well NUNC V-bottom polypropylene 0.45 ​ml plate (black) was used for this experiment. The constants *K*_*m*_ and *V*_max_ were calculated by fitting the data to the Michaelis-Menten equation by nonlinear regression.

### 2.6 Site-directed mutagenesis

To generate single mutants of *Toxo*Ddi1-RVP and *Cryp*Ddi1-RVP, site-directed mutagenesis was carried out with the QuickChange Site-Directed Mutagenesis kit (Stratagene-Agilten technologies, Inc., Santa Clare, CA, USA). Using this kit, a single amino acid residue, the catalytic residue Asp315 in *Toxo*Ddi1-RVP and Asp220 in *Cryp*Ddi1-RVP, was mutated to Ala (D315A/D220A) and Asn (D315N/D220N), and the mutations were confirmed by DNA sequencing. These four mutant proteins were expressed and purified in the same way as described for the wild-type protein. The purity of the mutants was checked by running 15% SDS-PAGE (Supplementary Fig. 1).

### 2.7 Crystallization

The microbatch method was employed for crystallization trials using the Hampton Research and Molecular Dimensions kits. 2 ​μl of the protein solution at 8–12 ​mg/ml concentration was mixed with 2 ​μl of various precipitants. The mixture was placed in the wells of the microbatch plate layered with paraffin and silicon oils (1:1) at 291 ​K. Crystals of *Toxo*Ddi1-RVP construct were obtained at a protein concentration of 8 ​mg/ml in a condition containing 1.8 ​M ammonium citrate tribasic (pH 7.0) and 30% 2-propanol. Crystals of *Cryp*Ddi1-RVP construct were obtained at a protein concentration of 8 ​mg/ml in a condition containing 0.1 ​M MES-sodium hydroxide (pH 6.0), 30% polyacrylate sodium salt and 10% ethanol. Octahedron-shaped crystals of *Toxo*Ddi1-RVP of length 0.1 ​mm and rectangular crystals of *Cryp*Ddi1-RVP of length 0.07 ​mm were obtained after 3–4 days.

### 2.8 Data collection

The X-ray diffraction data were collected from the crystals at XRD2 beamline using a Pilatus-6M detector at Elettra, Trieste, Italy, at a wavelength of 0.9918 ​Å. The crystals were cryoprotected with glycerol. The crystals of *Toxo*Ddi1-RVP diffracted to a resolution of 2.1 ​Å; 480 frames were collected with an oscillation angle of 0.5° per image and a crystal to detector distance of 250 ​mm. The crystals of *Cryp*Ddi1-RVP diffracted to a resolution of 2.8 ​Å; 360 frames were collected with an oscillation angle of 0.5° per image and a crystal to detector distance of 270 ​mm.

### 2.9 Model building and refinement

The data were processed and scaled using *iMOSFLM* and *AIMLESS* of the *CCP4* suite, respectively (Winn et al., 2011). The data collection and processing statistics are given in Table 1. The structures were determined by molecular replacement using *PHASER* (McCoy et al., 2007). The structures were manually built using *COOT* (Emsley and Cowtan, 2004) and refined by *REFMAC5* (Murshudov et al., 1997). The glycerol and water molecules were identified in the 2Fo-Fc and Fo-Fc electron density maps contoured at 1.0 and 3.0 σ levels, respectively. Several cycles of alternating model building and refinement were carried out until the *R*_work_ and *R*_free_ converged. Validation of the final structures was carried out using *MolProbity* (Chen et al., 2010) and the images were generated using *UCSF CHIMERA* (Pettersen et al., 2004). The atomic coordinates and structure factors were deposited in the Protein Data Bank with accession codes 7D66 for *Toxo*Ddi1-RVP and 7EFY for *Cryp*Ddi1-RVP, respectively.

**Table 1:** Data collection and refinement statistics. Values in parentheses are for the highest resolution shell.

	*Toxo*Ddi1-RVP (PDB id: 7D66)	*Cryp*Ddi1-RVP (PDB id: 7EFY)
**Wavelength (Å)**	0.99	0.99
**Resolution range (Å)**	65.27–2.13 (2.18-2.13)	47.44–2.80 (2.87-2.80)
**Space group**	*P*4_1_2_1_2	*H*32
**Unit cell (Å)**	*a* ​= ​*b* ​= ​184.60, *c* ​= ​184.34	*a* ​= ​*b* ​= ​81.55, *c* ​= ​142.18
**Total reflections**	471379 (32340)	35030 (5035)
**Unique reflections**	54712 (4404)	4686 (668)
**Multiplicity**	8.6 (7.3)	7.5 (7.5)
**Completeness (%)**	100 (100)	100 (100)
**<*I*/σ(*I*)>**	13.9 (3.1)	11.1 (2.1)
**Wilson *B*factor (Å**^**2**^**)**	42.16	81.30
***R*_m_**_**_erge_**_**(%)**	9.5 (67.8)	9.5 (10.0)
***R***_**meas**_**(%)**	10.4 (15.2)	10.9 (12.2)
***R*_p.i.m_(%)**	4.2 (67.8)	5.3 (6.0)
**CC**_**1/2**_	0.99 (0.37)	0.99 (0.84)
**Reflectionsused** in refinement	176969 (8844)	4683 (222)
***R***_**work**_**/*R***_**free**_	0.19/0.25	0.18/0.23
**RMSD bond (Å)**	0.0154	0.0064
**RMSD angles (ᵒ)**	1.833	1.497
**No. of atoms**		
**Protein**	11178	852
**Glycerol**	96	–
**Water**	585	5
**Ramachandran map**		
**Favored (%)**	95.93	100
**Allowed (%)**	4	–
**Outlier (%)**	0.07	–
**Average *B*factor (Å**^**2**^**)**		
**Protein**	34.26	90.50
**Glycerol**	47.25	–
**Water**	41.35	73.30

### 2.10 Molecular dynamics simulation and normal mode analysis (NMA)

An all-atom MD simulation of *Toxo*Ddi1-RVP dimer was carried out using *GROMACS 5.0.4* with amber 99SB force field (Hornak et al., 2006), to investigate the dynamics of the flap region. The protein was solvated using the TIP3P water model (Jorgensen et al., 1983) and kept in a box generated such that the minimum distance between the protein and the edge of the box was 1.0 ​nm. To neutralize the net charge, 0.1 ​M NaCl was added to the system. A distance cutoff of 10 ​Å was used for van der Waals interactions. Energy minimization was carried out using the steepest descent method. The system was equilibrated at NVT ensemble followed by NPT ensemble for 100 ps. Temperature equilibration was performed using a modified Berendsen thermostat (Berendsen et al., 1984) with a coupling time constant of 0.1 ps and a reference temperature of 300 ​K. Pressure equilibration was performed using the Parrinello-Rahman method (Parrinello and Rahman, 1981) with a coupling time constant of 2 ps and reference pressure of 1 ​bar. A time step of 2 fs was used in the leapfrog integrator. The simulation was carried out for a period of 300 ns and the coordinates and energies were recorded every 10 ps.

NMA analysis was carried out using the *elNemo* server (Suhre and Sanejouand, 2004).

### 2.11 Design of a modified peptide inhibitor

**Fig. 3:**
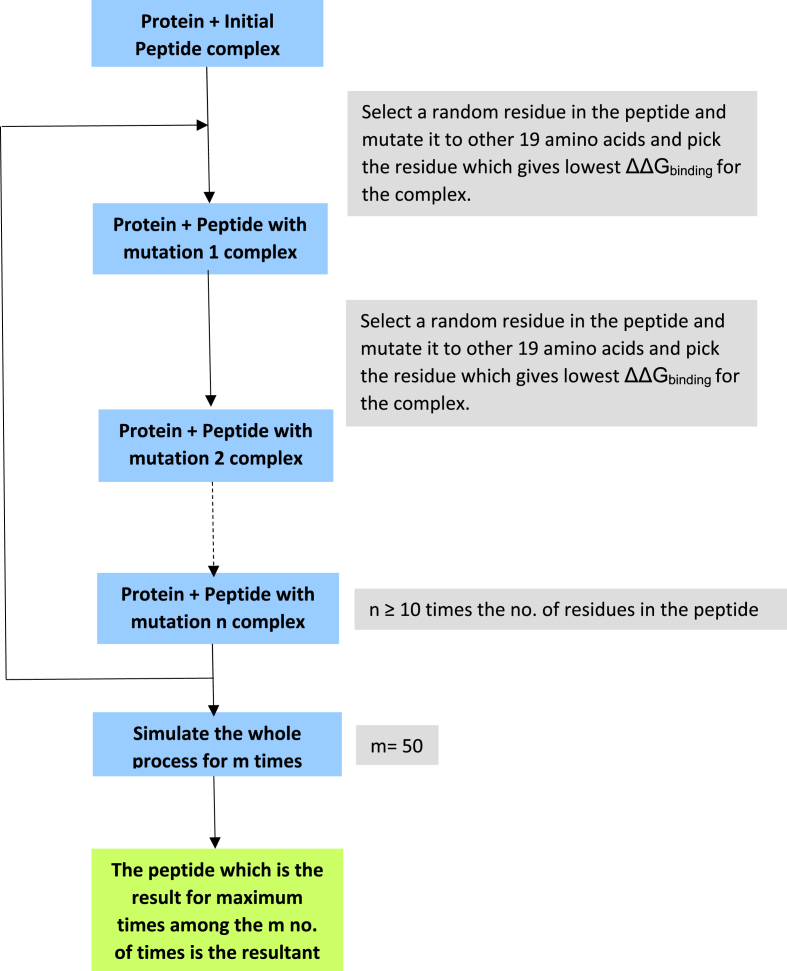
Computational workflow for the generation of a modified peptide.

The POS-SCAN module of the *FoldX* suite (Schymkowitz et al., 2005) was used to mutate every residue of the initial peptide (GVGRQEI) to all the nineteen other amino acid residues; the ΔΔG_binding_ value of the protein-peptide complex for each mutation was calculated. At a particular position, the mutation for which the ΔΔG_binding_ of the protein-peptide complex has the lowest value was considered. In the next step, a different residue of the peptide was selected randomly and mutated to all the nineteen other amino acid residues; the mutation for which the ΔΔG_binding_ of the protein-peptide complex has the lowest value was considered (Fig. 3). The same process was repeated multiple times to cover the whole peptide and obtain the lowest energy protein-peptide complex. This constituted one simulation of the process. Multiple simulations were performed to avoid reaching a local minimum of protein-peptide ΔG_binding_. The peptide which was the outcome of most of the simulations was considered to be the resultant peptide. The value of propensity for aggregation of the resultant peptide calculated using the *AGGRESCAN* (Conchillo-Solé et al., 2007) server suggested that the peptide was not prone to aggregation. The solubility of the peptide was checked using the *INNOVAGEN-PepCalc* tool.

### 2.12 Molecular docking

Molecular docking of *Toxo*Ddi1-RVP with nelfinavir, *Cryp*Ddi1-RVP with saquinavir and *Leish*Ddi1-RVP (PDB code: 5YQ8, Kumar and Suguna, 2018) with peptides was carried out using *AutoDock 4.2* (Morris et al., 2009). The coordinates of nelfinavir and saquinavir were extracted from the crystal structures of their complexes with HIV protease (PDB Codes: 2R5Q and 4QGI, respectively).

### 2.13 Sequence alignment

Multiple sequence alignments were carried out using *CLUSTAL Omega* (Sievers and Higgins, 2018). A structure-based sequence alignment was carried out using the *ESPript3* webserver (Gouet et al., 1999).

## 3 Results

### 3.1 Proteolytic activity of Ddi1 and its inhibition by HIV protease inhibitors

**Fig. 4:**
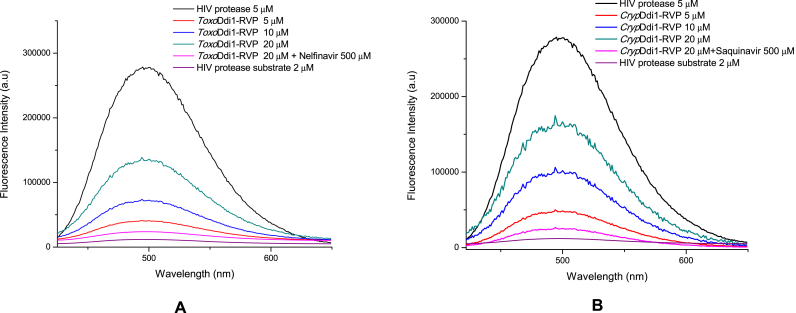
Fluorescence intensity profiles of (A) *Toxo*Ddi1-RVP and (B) *Cryp*Ddi1-RVP at pH 4.7 in the presence of HIV protease substrate-1.

Though the inhibitory effect of HIV protease inhibitors on the growth of parasitic protozoa has been studied, no direct evidence of the proteolytic activity of Ddi1 or the inhibitory effect of these inhibitors directly on Ddi1 through *in vitro* studies has been reported until now. We have carried out a FRET-based activity assay using the synthetic substrate, HIV Protease Substrate-1 (Sigma-aldrich) of the *Toxo*Ddi1-RVP and *Cryp*Ddi1-RVP constructs. An increase in the fluorescence intensity was observed with increasing protein concentration which implies an increase in the proteolytic cleavage of the substrate with increase in protein concentration. The fluorescence intensity did not increase for the reaction mixture containing 500 ​μM of the HIV protease inhibitor, nelfinavir with 20 ​μM of *Toxo*Ddi1-RVP (Fig. 4A) and for the reaction mixture containing 500 ​μM of the HIV protease inhibitor, saquinavir with 20 ​μM of *Cryp*Ddi1-RVP (Fig. 4B). These results indicate that nelfinavir has an inhibitory effect on the proteolytic activity of *Toxo*Ddi1-RVP and saquinavir has an inhibitory effect on the proteolytic activity of *Cryp*Ddi1-RVP as a result of which the substrate could not be cleaved.

**Fig. 5:**
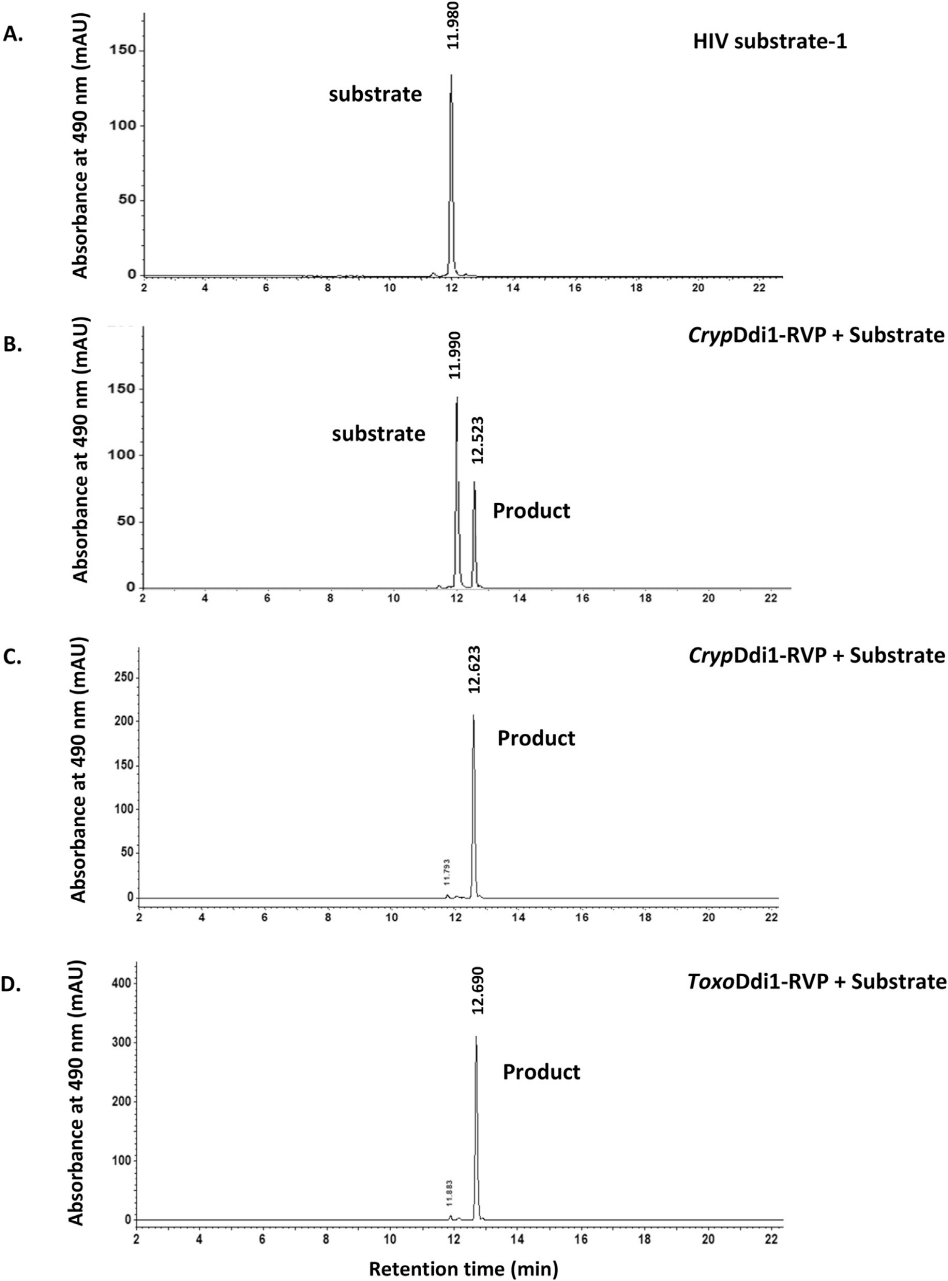
HPLC chromatogram for the proteolytic activity employing HIV protease substrate-1. (A) The substrate (B) *Cryp*Ddi1-RVP incubated with the substrate for 30 ​min showing the peaks of the substrate and the C-terminal fragment of the product while the reaction is in progress (C) *Cryp*Ddi1-RVP incubated with the substrate for 1 ​h showing the C-terminal fragment of the product and (D) *Toxo*Ddi1-RVP incubated with the substrate for 1 ​h showing the C-terminal fragment of the product.

**Fig. 6:**
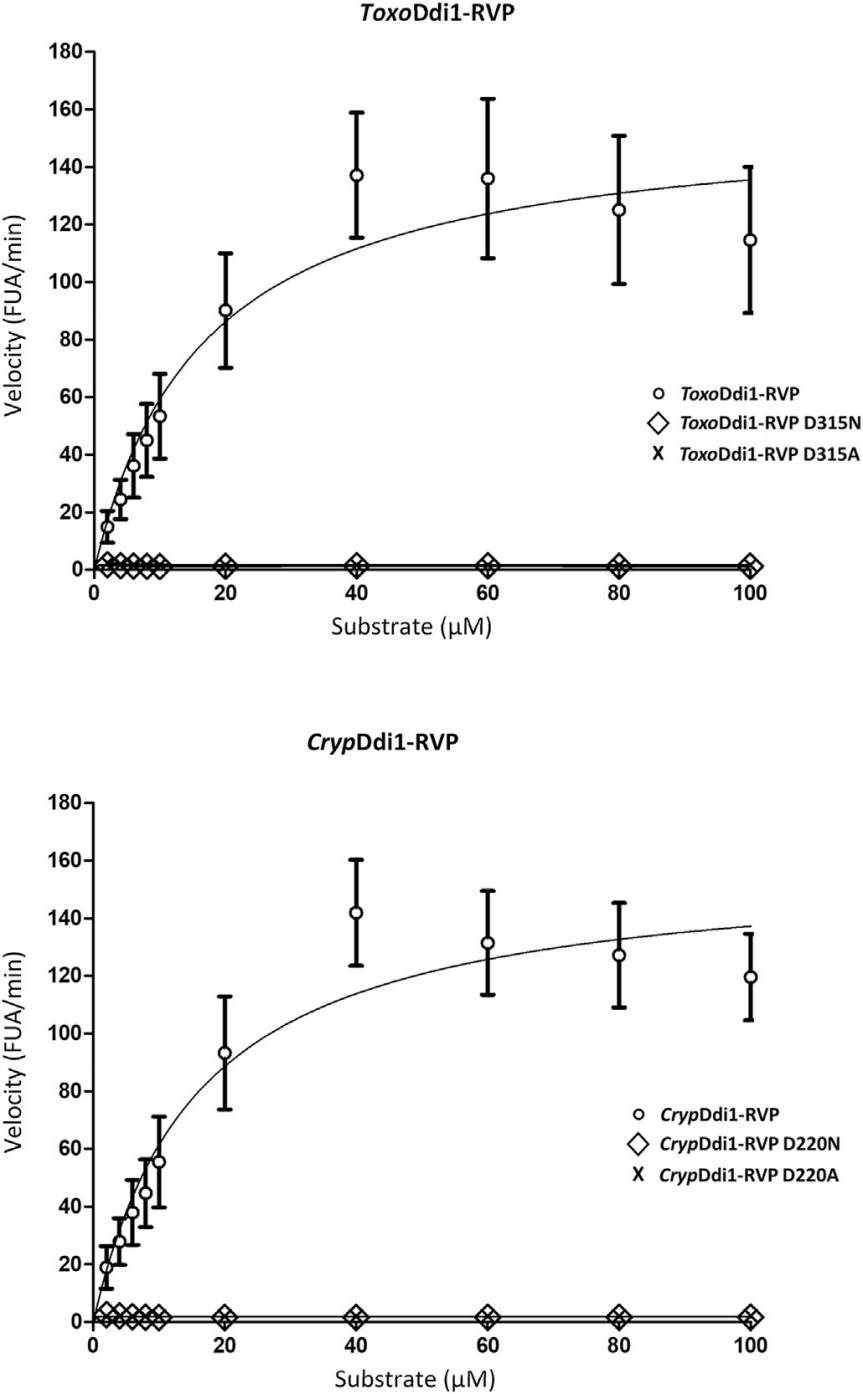
Kinetics of proteolysis of the HIV protease substrate-1 by *Toxo*Ddi1-RVP and *Cryp*Ddi1-RVP.

The HPLC experiments illustrated the cleavage of HIV protease substrate-1 by both *Toxo*Ddi1-RVP and *Cryp*Ddi1-RVP (Fig. 5). The substrate was completely cleaved and the labelled C-terminal peptide of the product appeared in the HPLC profile in the presence of these domains after 1 ​h of incubation. For *Cryp*Ddi1-RVP, both the substrate and the product were detected after an incubation period of 30 ​min, indicating that the reaction was in progress. The kinetics analysis (Fig. 6) showed a *V*_max_ of 158.30. ± 18.49 FUA/min and a *K*_*m*_ of 16.72 ​± ​5.95 ​μM for *Toxo*Ddi1-RVP and a *V*_max_ of 159.10 ​± ​14.85 FUA/min and a *K*_*m*_ of 15.94 ​± ​4.58 ​μM for *Cryp*Ddi1-RVP. Compared to these domains, the full length *Leish*Ddi1 displayed a better affinity with *V*_max_ and *K*_*m*_ values of 53.76 ​± ​1.60 FUA/min and 0.575 ​± ​0.288 ​μM, respectively (Perteguer et al., 2013).

Single mutants, D315A and D315N of *Toxo*Ddi1-RVP and D220A and D220N of *Cryp*Ddi1-RVP were generated (Supplementary Fig. 1) by mutating the catalytic residue Asp to Asn and Ala and activity assays were performed. As expected, no activity was observed (Fig. 6); this confirms the participation of Asp in the catalytic activity of these two domains as in other aspartic proteinases.

### 3.2 Binding of HIV protease inhibitors to Ddi1

**Fig. 7:**
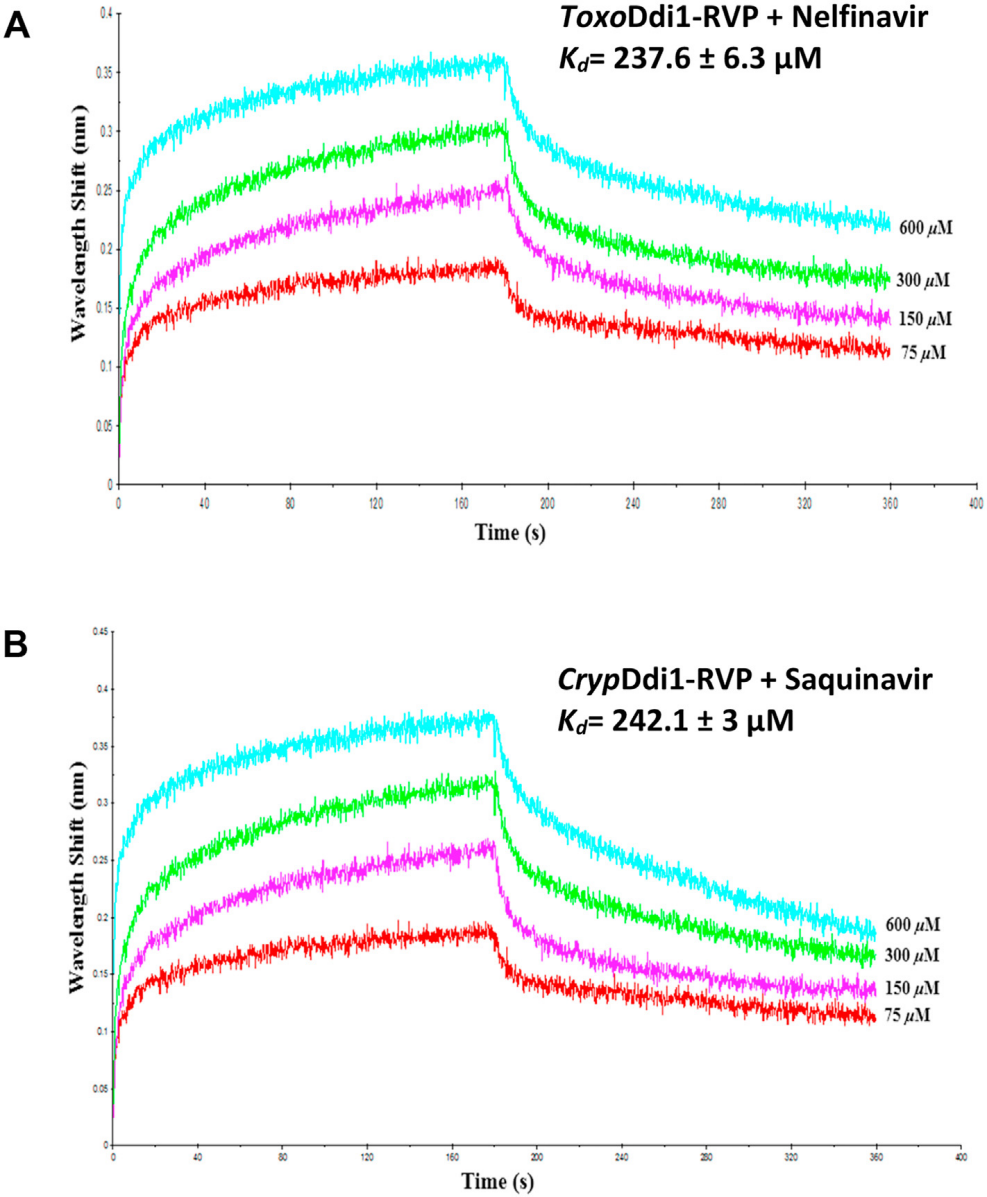
BLI binding profile of inhibitors with (A) *Toxo*Ddi1-RVP and (B) *Cryp*Ddi1-RVP.

In order to quantify the interaction of HIV protease inhibitors to Ddi1, we carried out *in vitro* binding studies of HIV protease inhibitors, saquinavir, nelfinavir, amprenavir, darunavir, indinavir and ritonavir to *Toxo*Ddi1-RVP and *Cryp*Ddi1-RVP using BLI. Of all the inhibitors tested, nelfinavir showed binding to *Toxo*Ddi1-RVP with a *K*_*d*_ of 237.6 ​± ​6.3 ​μM and saquinavir showed binding to *Cryp*Ddi1-RVP with a *K*_*d*_ of 242.1 ​± ​3 ​μM (Fig. 7 A,B, Supplementary Fig. 2). These affinities are slightly better than that for saquinavir binding to *Leish*Ddi1-fl with a *K*_*d*_ value of 314 ​± ​13 ​μM (Kumar and Suguna, 2018).

### 3.3 Binding of monoubiquitin and K48-linked diubiquitin to Ddi1

**Fig. 8:**
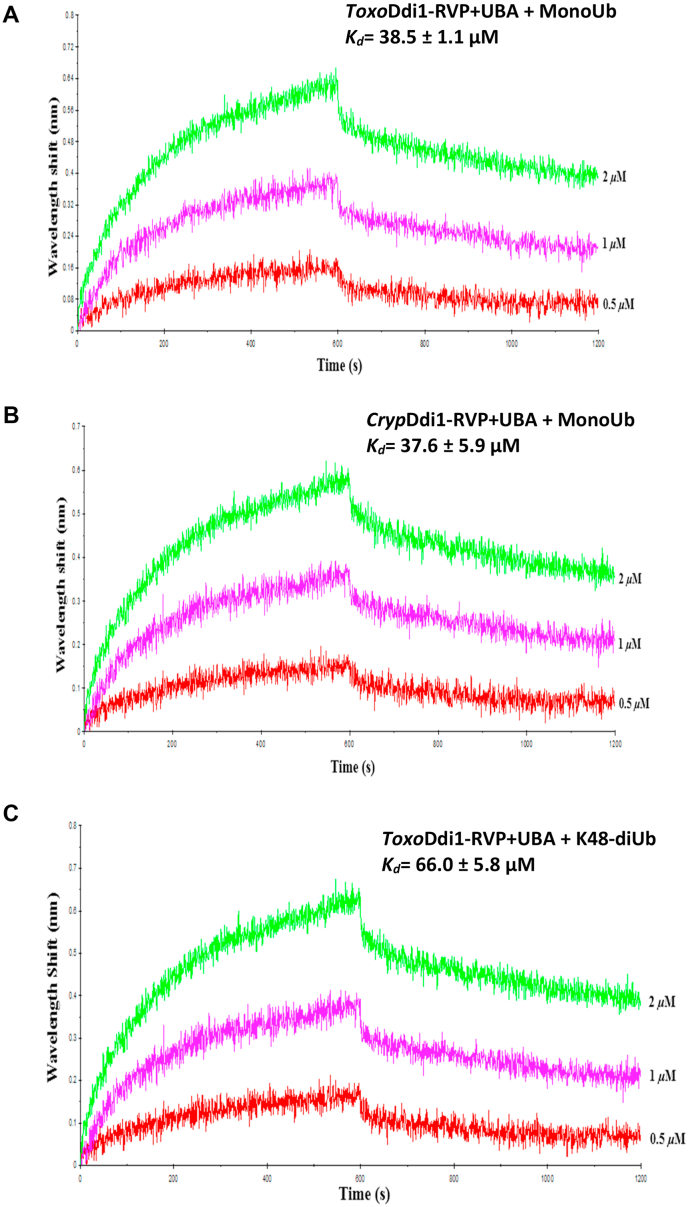
BLI binding profile of monoubiquitin with (A) *Toxo*Ddi1-RVP ​+ ​UB (B) *Cryp*Ddi1-RVP ​+ ​UBA and (C) K48-diubiquitin with *toxo*Ddi1-RVP ​+ ​UBA.

The binding of monoubiquitin and K48-linked diubiquitin to the UBA domain of yeastDdi1 was reported by Trempe et al. (2016). In order to check the binding of monoubiquitin and K48-linked diubiquitin to the UBA domain of Ddi1 from *T. gondii* and *C. hominis*, we carried out *in vitro* binding studies using BLI. Monoubiquitin showed binding to the *Toxo*Ddi1-RVP ​+ ​UBA construct with a *K*_*d*_ of 38.5 ​± ​1.1 ​μM (Fig. 8A) and *Cryp*Ddi1-RVP ​+ ​UBA with a *K*_*d*_ of 37.6 ​± ​5.9 ​μM (Fig. 8B). These values are comparable to the *K*_*d*_ value of 43 ​± ​6 ​μM for monoubiquitin binding to the yeastDdi1-RVP ​+ ​UBA construct (Trempe et al., 2016). The K48-linked diubiquitin showed binding to the*Toxo*Ddi1-RVP ​+ ​UBA construct with a *K*_*d*_ of 66 ​± ​5.8 ​μM (Fig. 8C), while it did not show binding to *Cryp*Ddi1-RVP.

### 3.4 Structure determination

**Fig. 9:**
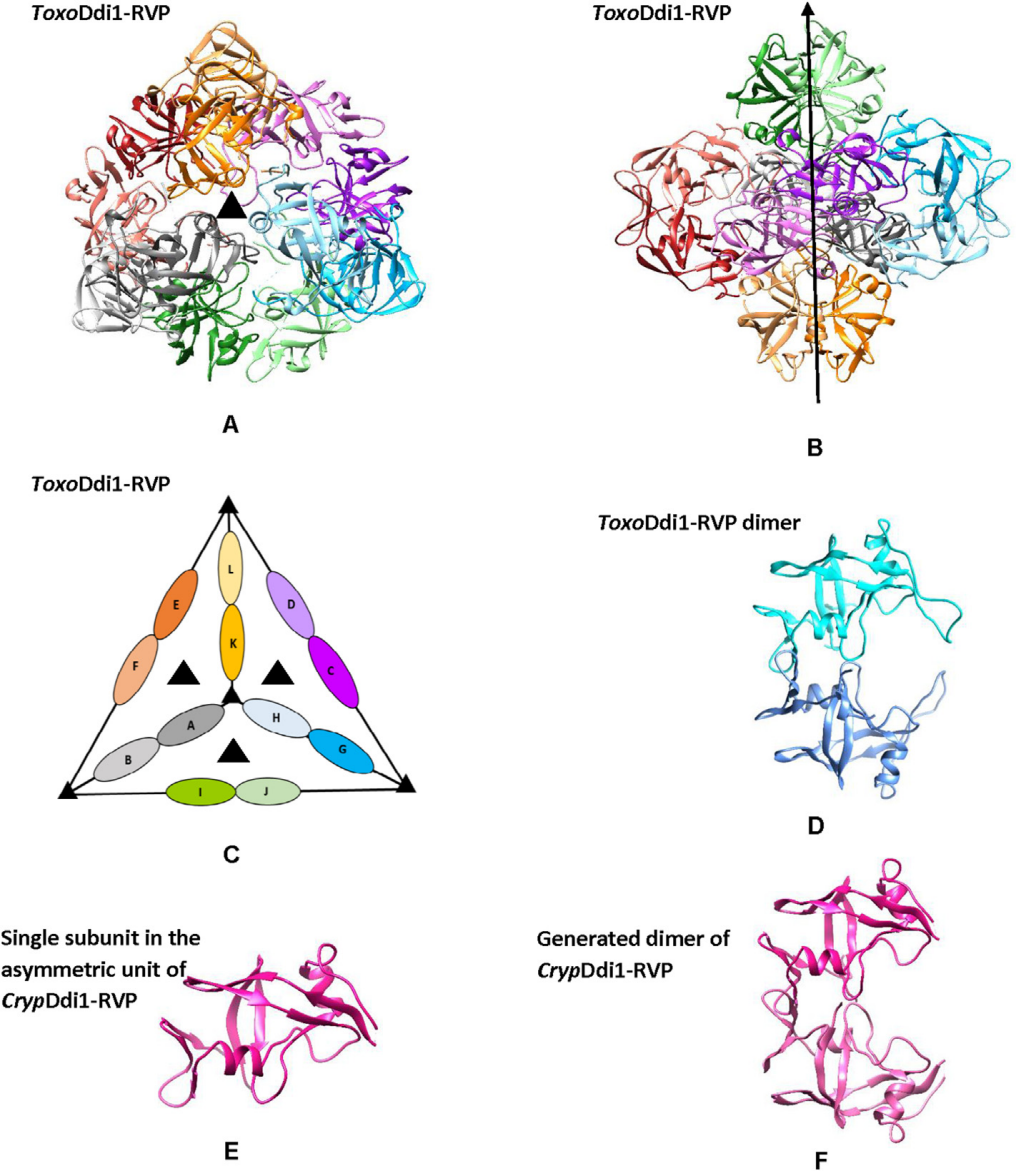
Crystal structures. In *Toxo*Ddi1-RVP **(**A) Arrangement of molecules down the 3-fold axis (B) About the 2-fold axis (C) Schematic representation of the arrangement of dimers in the ASU (D) The dimer. In *Cryp*Ddi1-RVP (E) One molecule in the ASU and (F) The generated dimer upon application of crystallographic symmetry.

Crystallization trials for three constructs of Ddi1 from *T. gondii*, *Toxo*Ddi1-full, *Toxo*Ddi1-RVP ​+ ​UBA and *Toxo*Ddi1-RVP and three constructs of Ddi1 from *C. hominis*, *Cryp*Ddi1-full, *Cryp*Ddi1-RVP ​+ ​UBA and *Cryp*Ddi1-RVP were carried out. Crystals were obtained for the constructs *Toxo*Ddi1-RVP and *Cryp*Ddi1-RVP. The structure of *Toxo*Ddi1-RVP was determined by molecular replacement using the yeastDdi1-RVP structure (PDB Code: 2I1A) as the search model which has 45% sequence identity with *Toxo*Ddi1-RVP. The solution had six dimers in the asymmetric unit (ASU) (Fig. 9A) with TFZ and LLG of 35 and 3173, respectively, in the space group *P*4_1_2_1_2. The following non-crystallographic symmetries relate the subunits in the ASU: (i) 2-fold symmetry that related the two halves of the ASU (Fig. 9B), (ii) 3-fold symmetry relates the three dimers on each face of the tetrahedron (Fig. 9C) and (iii) 2-fold symmetry that relates the two subunits of the dimers (Fig. 9D). Out of the six dimers in the ASU, the flap regions of both the subunits in only one dimer and the flaps of three other subunits in different dimers had electron density. The structure of *Cryp*Ddi1-RVP was determined by molecular replacement using the *Toxo*Ddi1-RVP structure as the search model which has 61% sequence identity with *Toxo*Ddi1-RVP. The solution had one molecule in the ASU (Fig. 9E) with TFZ and LLG of 29 and 899, respectively, in the space group *H*32. The electron density corresponding to the flap region was missing. The dimer could be generated upon application of the crystallographic 2-fold symmetry (Fig. 9F).

### 3.5 The structural fold

**Fig. 10:**
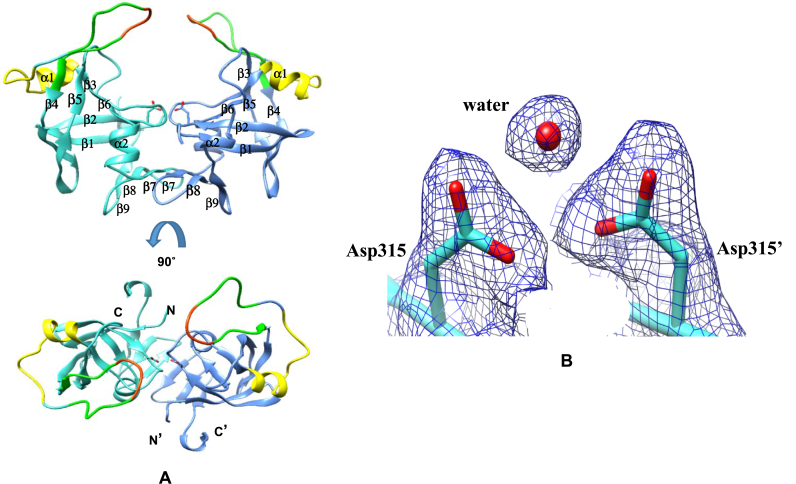
Structure of *Toxo*Ddi1-RVP. (A) The dimer showing the flap in green (residues 341–356), tip of the flap in red (residues 347–350), and flap elbow in yellow (residues 325–338). Catalytic aspartates are shown as sticks and (B) 2|F_O_|-|F_C_| electron density map contoured at 1σ showing water molecule at the active site. (For interpretation of the references to colour in this figure legend, the reader is referred to the Web version of this article.)

The fold and the active site architecture of *Toxo*Ddi1-RVP and *Cryp*Ddi1-RVP are similar to those of HIV protease. A structure-based sequence alignment of the RVP domains is shown in Supplementary Fig. 3. *Toxo*Ddi1-RVP and *Cryp*Ddi1-RVP form homodimers where each subunit is predominantly made up of β-sheets. Two aspartates, one from each subunit form the catalytic site at the dimer interface. An interdomain β-sheet is formed by three strands from each domain (Fig. 10A). As observed in other aspartyl protease structures, a water molecule is present within the hydrogen bonding distance of the active site aspartates in the *Toxo*Ddi1-RVP structure (Fig. 10B).

### 3.6 The active site

The active site is located at the dimer interface in the cleft between the two subunits of the dimer. The motif DSG, containing the catalytic aspartate, is present at the tip of a loop called the ψ loop in each polypeptide chain in the active site. As observed in HIV protease and other pepsins, a network of hydrogen bonds at the dimeric interface, called the “fireman's grip” stabilizes the active site in *Toxo*Ddi1-RVP (Supplementary Fig. 4A) and *Cryp*Ddi1-RVP. The hydrophobic-hydrophobic-glycine (HHG) motif is a conserved structural feature of all aspartic proteases. In *Toxo*Ddi1-RVP and *Cryp*Ddi1-RVP, it is formed by Leu-Phe-Gly. In *Toxo*Ddi1-RVP as well as in *Cryp*Ddi-RVP, the backbone nitrogen atoms of the catalytic aspartates are hydrogen bonded to the backbone carbonyl oxygen of Phe residue in the HHG motif (Supplementary Fig. 4B).

In the *Toxo*Ddi1-RVP and *Cryp*Ddi1-RVP structures, the substrate binding groove is lined mainly by hydrophobic amino acid residues suggesting that they recognize the substrate via hydrophobic interactions. In the *Toxo*Ddi1-RVP structure, a hydrophobic binding cavity is formed by the side chains of residues Met398, Ph305, Phe313, Ala318, Phe322, Ile353, Thr374, Leu376, Val381 and Leu384, (Supplementary Fig. 4C). Similarly in the *Cryp*Ddi1-RVP structure, the side chains of residues Met203, Tyr205, Phe218, Ala223, Ile227, Ile258, Thr279, Leu281, Val286 and Leu289 form a hydrophobic binding cavity. In both the cases, the catalytic Asp residues form an acidic patch at the centre of the cavity. The binding cavity in the *Toxo*Ddi1-RVP structure has a width of ∼27 ​Å (distance between the Cα atoms of Asn379 residues of the two subunits of the dimer), while in the *Cryp*Ddi1-RVP structure, the cavity width is ∼22.5 ​Å (distance between the Cα atoms of Ser284 of the two subunits) (Supplementary Fig. 4D). The cavity in *Toxo*Ddi1-RVP is larger than that formed in HIV protease which has a width of ∼19–22 ​Å in the open form, while the size of the cavity in *Cryp*Ddi1-RVP is comparable to that of HIV protease.

### 3.7 The flap region

**Fig. 11:**
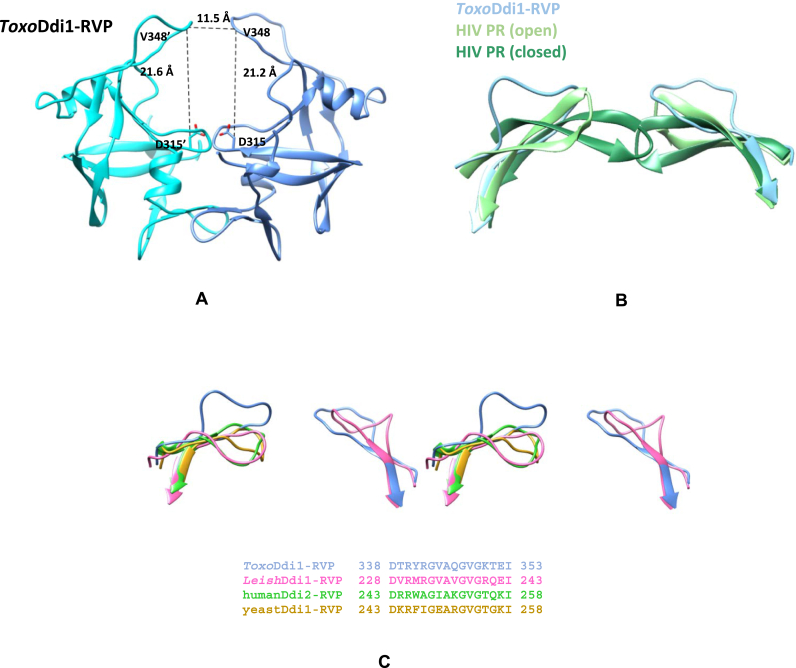
Flap region of Ddi1-RVP. (A) Distances between the Cα atoms of Val348 and Val348’; Cα of Val348 and Cβ of Asp315 in *Toxo*Ddi1-RVP (B) Superposition of the flaps of *Toxo*Ddi1-RVP (blue), HIV protease in open form (light green) and HIV protease in closed form (dark green) (C) A stereo view of the superposition of the flaps of *Toxo*Ddi1-RVP (blue), *Leish*Ddi1-RVP (pink), humanDdi2-RVP (green) and yeastDdi1-RVP (yellow) and their sequences. (For interpretation of the references to colour in this figure legend, the reader is referred to the Web version of this article.)

In HIV protease, an extended loop called the ‘flap’ covers the active site, has a β-hairpin structure and is known to stabilize the binding of the substrate or the inhibitor. The flap of *Toxo*Ddi1-RVP forms a loop without the characteristic β-hairpin structure (Fig. 11A). The distance between the Cα atoms of Val348 residues located at the tips of the two flaps is 11.5 ​Å. The flaps are equidistant from the active site, as in HIV protease. The distance between the Cα atom of Val348 and the Cα atom of Asp315 (catalytic aspartate) is 21.6 ​Å in one subunit and 21.2 ​Å in the other. In the *Cryp*Ddi1-RVP structure, ten residues of the flap region could not be traced.

**Fig. 12:**
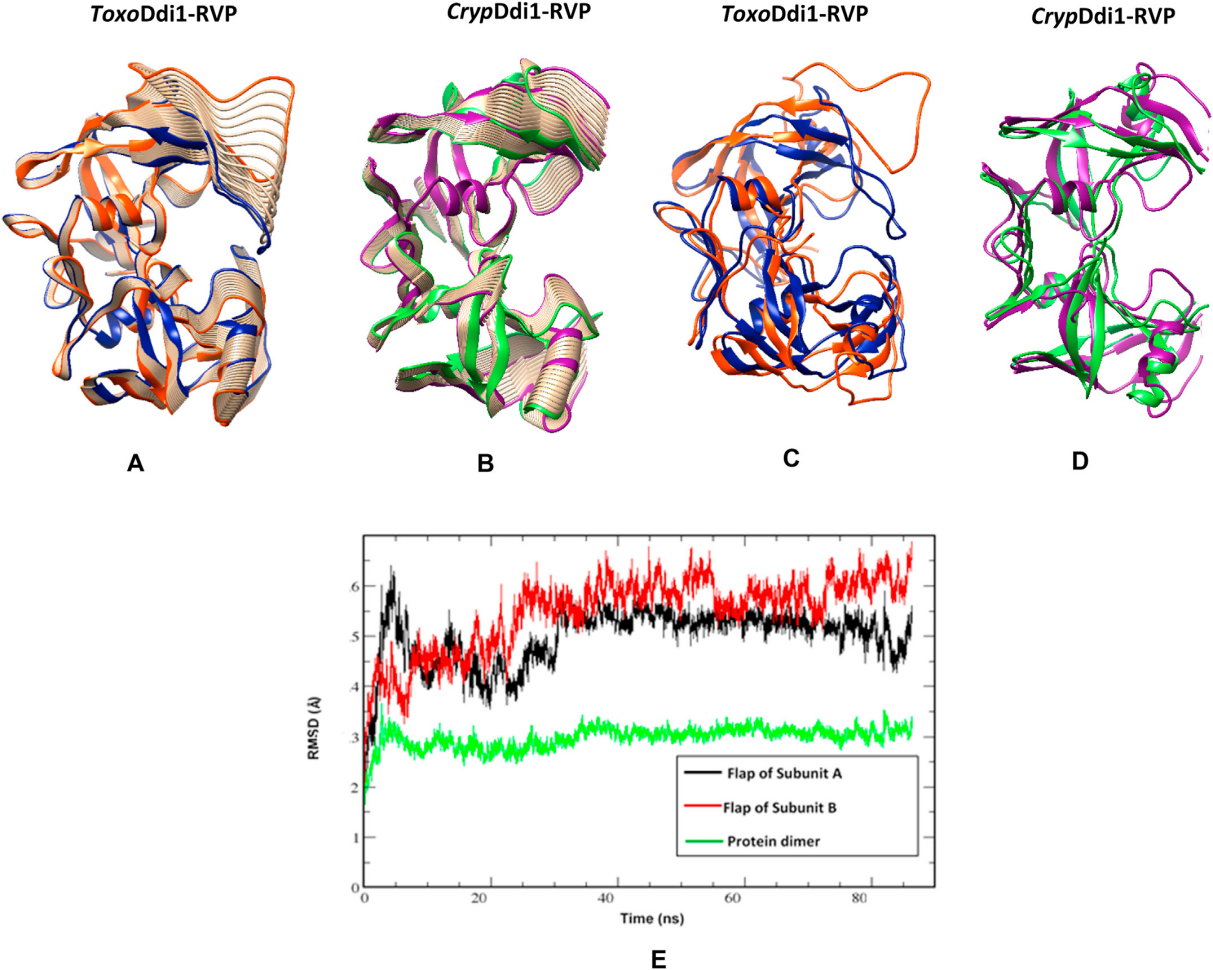
Normal mode analysis. Movement of (A) *Toxo*Ddi1-RVP and (B) *Cryp*Ddi1-RVP captured by NMA. Superposition of (C) Open (orange) and closed (blue) forms of *Toxo*Ddi1-RVP and (D) Open (green) and closed (purple) forms of *Cryp*Ddi1-RVP and (E) RMSD plot of Cα atoms for *Toxo*Ddi1-RVP for the last 85 ns.

In HIV protease, the flap closes inwards upon substrate/inhibitor binding at the active site, assuming a closed conformation in contrast to the open or semi-open conformation in the inhibitor-free form (Fig. 11B). The two subunits were also observed to move closer to each other due to an overall conformational change. The width of the binding cavity changes from 21.9 ​Å (distance between the Cα atoms of Pro81 in the dimer) in its open form (PDB code: 3HVP), to 18.9 ​Å in its closed form (PDB code: 4HVP). Superposition of the structures of *Toxo*Ddi1-RVP, *Leish*Ddi1-RVP (PDB code: 5YQ8), yeastDdi1-RVP (PDB code: 4Z2Z) and humanDdi2-RVP (PDB code: 4RGH) reveals that the flaps of *Toxo*Ddi1-RVP and *Leish*Ddi1-RVP are in a more open conformation compared to those in the other two. Total conservation of half of the residues was observed when the sequences of these four flap regions were compared (Fig. 11C). Variations in the other half of the residues perhaps results in the flaps having different conformations, especially at the tips of the flaps. As a result, the width of the binding pockets varies within 5 ​Å in these RVP domains. This indicates that though the overall architecture of the binding pocket is retained, there are significant differences in the detailed features suggesting that the corresponding substrates can be similar but not exactly the same. To investigate the structural flexibility of *Toxo*Ddi1-RVP and *Cryp*Ddi1-RVP, a normal mode analysis (NMA) was carried out using the *elNemo* server (Suhre and Sanejouand, 2004) that uses an elastic network model to generate movements in biological macromolecules that are functionally relevant. In this study, a total of 16 modes were generated and analyzed in each structure. One of the modes in each case showed significant flap movements from the open to the closed forms and movement of the entire protein to a lesser extent, leading to a change in the size of the binding cavity, as in the case of HIV protease (Fig. 12A–D).

In retropepsins, the sequence of the flap is known to play a crucial role in its dynamics and inhibitor binding. The binding affinity of HIV protease inhibitors decreased when the residues in the flap region were mutated (Yu et al., 2015). Only two glycine residues at the tip of the flap regions are conserved between the sequences of the flaps of *Toxo*Ddi1-RVP and HIV protease. The dynamics of the flap and the nature of the substrate it binds to will depend on the sequence of the flap. An all-atom MD simulation of *Toxo*Ddi1-RVP was carried out for 300 ns, to obtain insights into the extent of flexibility and movement of the flap region. During the simulation, the dimer remained stable and did not deviate significantly from the crystal structure. The root mean square deviation (RMSD) value of Cα atoms between the structures generated over the course of simulation and the crystal structure was about 3 ​Å. The flaps of both the subunits showed significant movement. The MD simulations showed that RMSD values of the flaps of subunits A and B stabilized at about 5.5 and 6.5 ​Å, respectively (Fig. 12E). This indicates a slight asymmetric behavior in the dynamics of the flaps in the dimer.

### 3.8 Docking of HIV protease inhibitors

**Fig. 13:**
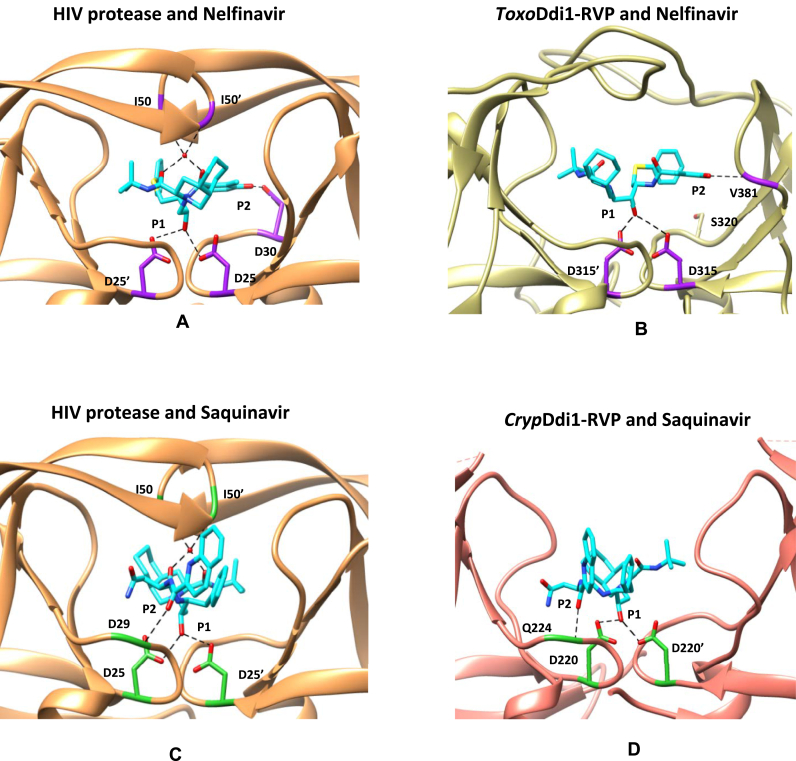
Complexes with inhibitors. (A) HIV protease with nelfinavir (PDB code: 2R5Q; Coman et al., 2008) (B) *Toxo*Ddi1-RVP with nelfinavir in docked conformation (interacting residues in purple) (C) HIV protease with saquinavir (PDB code: 4QGI; Goldfarb et al., 2015) and (D) *Cryp*Ddi1-RVP with saquinavir in docked conformation (interacting residues in green). (For interpretation of the references to colour in this figure legend, the reader is referred to the Web version of this article.)

Several attempts to crystallize *Toxo*Ddi1-RVP in complex with nelfinavir and *Cryp*Ddi1-RVP in complex with saquinavir were unsuccessful. To obtain insights into possible interactions between Ddi1-RVP and inhibitors, we carried out molecular docking studies. Nelfinavir docked to *Toxo*Ddi1-RVP with a binding energy of −7.2 ​kcal/mol in a manner similar to that observed in the crystal structure of the complex of HIV protease with nelfinavir. A comparison of the docking results with the crystal structure of HIV protease in complex with nelfinavir (PDB code: 2R5Q; Coman et al., 2008), reveals that the interaction between the hydroxyl group at the P1 position of nelfinavir and the carboxyl group of the active site aspartates is retained (Fig. 13A). The hydroxyl group at the P2 position of the inhibitor that interacts with the side chain of Asp30 in the case of HIV protease, interacts with the backbone –NH of Val381 of one subunit of the *Toxo*Ddi1-RVP dimer (Fig. 13B). Saquinavir docked to *Cryp*Ddi1-RVP with a binding energy of −6.5 ​kcal/mol in a similar manner as observed in the crystal structure of the complex of HIV protease with saquinavir. A comparison of the docking results of saquinavir to *Cryp*Ddi1-RVP with the crystal structure of HIV protease in complex with saquinavir (PDB code: 4QGI; Goldfarb et al., 2015), reveals that the interaction between the hydroxyl group at the P1 position of saquinavir and the carboxyl groups of the active site aspartates is retained in the docked structure. The hydroxyl group at the P2 position of the inhibitor that interacts with the side chain of Asp29 in the case of HIV protease (Fig. 13C) interacts with the backbone –NH of Gln224 of *Cryp*Ddi1-RVP (Fig. 13D).

### 3.9 Binding and docking studies of the initial and the modified peptides to the Ddi1-RVP from *L. major*

**Fig. 14:**
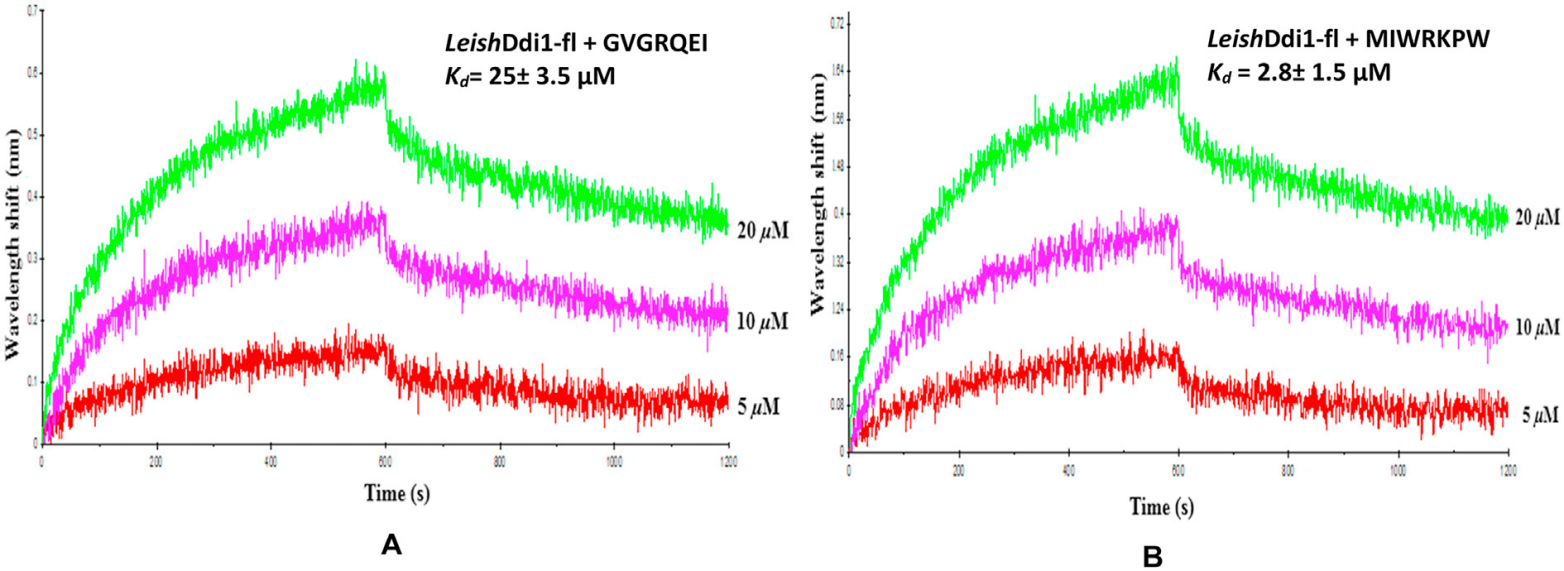
BLI binding profile of (A) Initial peptide (GVGRQEI) and (B) Modified peptide (MIWRKPW) with *Leish*Ddi1-full length protein.

**Fig. 15:**
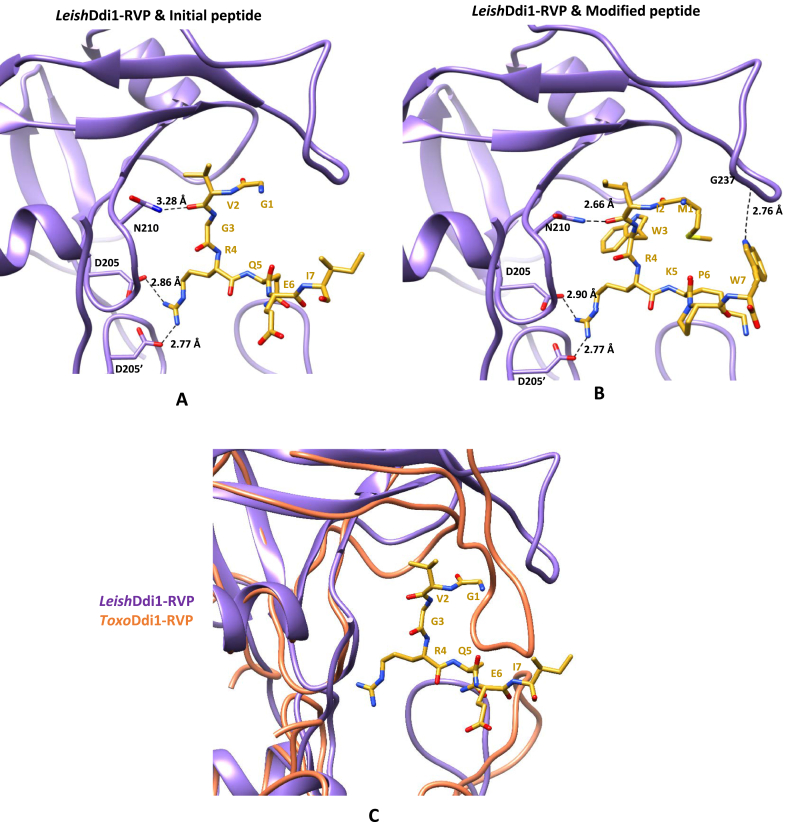
Active site interactions of *Leish*Ddi1-RVP (PDB code: 5YQ8, Kumar and Suguna, 2018) with peptide inhibitors. (A) Initial peptide (GVGRQEI) (B) Modified peptide (MIWRKPW) and (C) Superposition of *Toxo*Ddi1-RVP (orange) on *Leish*Ddi1-RVP (purple) with the initial peptide. (For interpretation of the references to colour in this figure legend, the reader is referred to the Web version of this article.)

The initial peptide (GVGRQEI) showed binding to the protein with a *K*_*d*_ of 25.3 ​± ​3.5 ​μM (Fig. 14A), while, the modified peptide (MIWRKPW) showed almost ten times better binding to the protein with a *K*_*d*_ of 2.8 ​± ​1.5 ​μM (Fig. 14B). Docking studies were performed for a better understanding of the binding modes of the initial and modified peptides to the protein. In one of the docked conformations of the initial peptide, it was observed that it docks to the protein in an orientation similar to that observed in the crystal structure, retaining the strong ionic interaction of the Arg residue of the peptide with Asp205 and Asp205′ at the active site of the protein and also the hydrogen bonding interaction of the main chain carbonyl oxygen atom of the Val residue of the peptide with the side chain of Asn210 of the protein (Fig. 15A). For the docked conformation of the initial peptide and the protein, the binding energy is −4.5 ​kcal/mol. In the docked structure of the modified peptide to *Leish*Ddi1-RVP, it was observed that the interaction of the Arg residue to Asp205 and Asp205’ is retained along with the interaction of the main chain carbonyl oxygen atom of the Ile residue of the peptide to the side chain of Asn210 of the protein. In addition, another hydrogen bonding interaction between the side chain of the Trp residue of the peptide and the main chain carbonyl oxygen atom of Gly237 in the flap region of the protein was observed (Fig. 15B). The binding energy is −6.6 ​kcal/mol for the docked conformation of the modified peptide and protein. The peptides did not show any binding to *Toxo*Ddi1-RVP and *Cryp*Ddi1-RVP as in *Toxo*Ddi1-RVP, since the flap region makes short contacts with the initial peptide as has been observed upon superposition of *Toxo*Ddi1-RVP on the *Leish*Ddi1-RVP structure (Fig. 15C).

## 4 Discussion

HIV protease inhibitors were found to reduce the severity of protozoal infections in AIDS patients. Subsequent studies showed that the inhibitors target the Ddi1-RVP domain (White et al., 2011). More studies on the structure and dynamics of Ddi1 and its activity and inhibitor binding may direct further investigations towards the design of better antiprotozoal compounds. In HIV protease, the substrate/inhibitor binding is strengthened as the subunits move closer to each other and the flap closes over the binding cavity. However, it is not clear whether the Ddi1-RVP domain behaves in a similar manner or its structural framework is too rigid to bind to HIV protease inhibitors. The present study on *Toxo*Ddi1-RVP and *Cryp*Ddi1-RVP domains aims to address these points. The crystal structures of *Toxo*Ddi1-RVP and *Cryp*Ddi1-RVP show that they share several structural features such as the fold, the active site architecture, and the hydrophobic nature of the substrate binding cavity, with HIV protease. The binding cavities of *Toxo*Ddi1-RVP, yeastDdi1-RVP (Sirkis et al., 2006) and *Leish*Ddi1-RVP (Kumar and Suguna, 2018) are similar in size but larger compared to that of HIV protease. In addition, there are variations in the binding site residues. Instead of Asp29, Asp30, and Val82, which interact with inhibitors in HIV protease, *Toxo*Ddi1-RVP has Gln319, Ser320, and Glu382, and *Cryp*Ddi1-RVP has Gln224, Thr225, and Asp287, respectively. Due to these small but subtle variations, the nature of the inhibitors may be different as the surface charge and polarity of the binding site are altered. Further, contrary to the case in HIV protease, the flap of *Toxo*Ddi1-RVP has no secondary structure. The flap regions in Ddi1-RVP appear to be highly mobile as is evident from the missing electron density for one of the flaps in yeastDdi1-RVP and humanDdi2-RVP and for both the flaps in *Cryp*Ddi1-RVP. The electron density for the flaps of both the subunits of one dimer is visible in *Toxo*Ddi1-RVP. The larger binding cavity and the presence of such a flexible flap suggest that Ddi1-RVP may accommodate bigger substrates. Nrf1 was proposed to be the natural substrate of human Ddi2 (Koizumi et al., 2016); however, protozoa do not have any homologues of Nrf1, implicating that protozoal Ddi1 will target entirely different molecules. Further, the NMA analysis that we carried out to explore possible conformational changes and modulation of the binding pocket due to inhibitor binding revealed the structural flexibility of *Toxo*Ddi1-RVP and *Cryp*Ddi1-RVP dimers similar to that of HIV protease. The binding affinities of *Toxo*Ddi-RVP and *Cryp*Ddi1-RVP for HIV protease inhibitors are in the micromolar range (compared to nanomolar values for HIV protease) in solution as suggested by BLI studies. The MD simulation studies carried out on *Toxo*Ddi1-RVP reveal that the flaps are highly flexible. Also, the flaps of the two subunits of the dimer show a slightly asymmetric behaviour in terms of their dynamics. The peptide inhibitor proposed from the crystal structure of *Leish*Ddi1-RVP shows binding to the protein through BLI studies and the modified peptide generated shows better binding than the initial peptide as indicated by their *K*_*d*_ values.

Our study demonstrates the proteolytic activity of the isolated Ddi1-RVP domain for the first time. YeastDdi1 was shown to act on polyubiquitinated (more than eight chains) substrates (Yip et al., 2020) but the cleavage of peptides by neither the full length Ddi1 (Trempe et al., 2016) nor the isolated RVP domain was detected. Both the UBL and the HDD domains were found to be essential for the binding of the substrates. No activity was detected for humanDdi2 or its RVP domain (Sivá et al., 2016). Proteolytic activity (*in vitro*) was detected only for *Leish*Ddi1-fl using the synthetic HIV protease peptide substrate-1 at pH 5 (Perteguer et al., 2013). While the presence of HDD and the similarities in the structures and the active site architecture suggest that *Toxo*Ddi1-RVP may have the capability to cleave ubiquitin and the substrates upon polyubiquitination similar to the yeastDdi1, in the case of *Cryp*Ddi1-RVP as the HDD domain is missing, the triggering mechanism has to be different and is yet to be investigated. Subtle but significant changes in the geometry and residues in the substrate binding cavities of the RVP domains and marked differences in the domain organization of the full length proteins in different organisms suggest that the corresponding substrates and/or cleavage sites could vary considerably while sharing some common features.

For a long time after protection against protozoal infections by HIV protease inhibitors was detected, efforts for the development of more effective drugs did not progress well, as the target for these inhibitors was not known. Though Ddi1 was later identified as a possible target, there is a dearth of structural information on Ddi1. The present study on the crystal structures of the RVP domains of *Toxo*Ddi1 and *Cryp*Ddi1 contributes to expand the available structural data base on these proteins. Though HIV protease inhibitors showed weak binding, it is an encouraging result and provides a starting point for further improvements and an alternate strategy to the pursuit to develop specific inhibitors to Ddi1 in protozoa.

## Funding sources

This work was supported by a grant from the Department of Biotechnology (10.13039/501100001407DBT), Government of India and funds from the DBT-IISc Partnership Program for Advanced Research in Biological Sciences and Bioengineering.

## CRediT authorship contribution statement

**Killivalavan Asaithambi:** Formal analysis, Writing – original draft. **Iman Biswas:** Formal analysis, Writing – original draft. **Kaza Suguna:** Formal analysis, Writing – original draft.

## Declaration of competing interest

The authors declare that they have no known competing financial interests or personal relationships that could have appeared to influence the work reported in this paper.

## References

[bib1] Aufderheide A., Unverdorben P., Baumeister W., Förster F. (2015). Structural disorder and its role in proteasomal degradation. FEBS Lett..

[bib2] Berendsen H.J.C., Postma J.P.M., Van Gunsteren W.F., Dinola A., Haak J.R. (1984). Molecular dynamics with coupling to an external bath. J. Chem. Phys..

[bib3] Bouvier L.A., Niemirowicz G.T., Salas-Sarduy E., Cazzulo J.J., Alvarez V.E. (2018). DNA-damage inducible protein 1 is a conserved metacaspase substrate that is cleaved and further destabilized in yeast under specific metabolic conditions. FEBS J..

[bib4] Chen V.B., Arendall W.B., Headd J.J., Keedy D.A., Immormino R.M., Kapral G.J., Murray L.W., Richardson J.S., Richardson D.C. (2010). *MolProbity*: all-atom structure validation for macromolecular crystallography. Acta Crystallogr. D Biol. Crystallogr..

[bib5] Clarke D.J., Mondesert G., Segal M., Bertolaet B.L., Jensen S., Wolff M., Henze M., Reed S.I. (2001). Dosage suppressors of pds1 implicate ubiquitin-associated domains in checkpoint control. Mol. Cell Biol..

[bib6] Coman R.M., Robbins A.H., Fernandez M.A., Gilliland C.T., Sochet A.A., Goodenow M.M., McKenna R., Dunn B.M. (2008). The contribution of naturally occurring polymorphisms in altering the biochemical and structural characteristics of HIV-1 subtype C protease. Biochemistry.

[bib7] Conchillo-Solé O., de Groot N.S., Avilés F.X., Vendrell J., Daura X., Ventura S. (2007). AGGRESCAN: a server for the prediction and evaluation of "hot spots" of aggregation in polypeptides. BMC Bioinf..

[bib8] Emsley P., Cowtan K. (2004). Coot: model-building tools for molecular graphics. Acta Crystallogr. Sect. D Biol. Crystallogr..

[bib9] Fassmannova D., Sedlák F., Sedláček J., Špička I., Šašková K.G. (2020). Nelfinavir inhibits the TCF11/Nrf-1 mediated proteasome recovery pathway in myeloma. Cancers.

[bib10] Gabriely G., Kama R., Gelin-Licht R., Gerst J.E. (2008). Different domains of the UBL-UBA ubiquitin receptor, Ddi1/Vsm1, are involved in its multiple cellular roles. Mol. Biol. Cell.

[bib11] Goldfarb N.E., Ohanessian M., Biswas S., McGee T.D., Mahon B.P., Ostrov D.A., Garcia J., Tang Y., McKenna R., Roitberg A., Dunn B.M. (2015). Defective hydrophobic sliding mechanism and active site expansion in HIV-1 protease drug resistant variant Gly48Thr/Leu89Met: mechanisms for the loss of saquinavir binding potency. Biochemistry.

[bib12] Gouet P., Courcelle E., Stuart D.I., Métoz F. (1999). ESPript: analysis of multiple sequence alignments in PostScript. Bioinformatics.

[bib13] Gu Y., Wang X., Wang Y., Wang Y., Li J., Yu F.-X. (2020). Nelfinavir inhibits human Ddi2 and potentiates cytotoxicity of proteasome inhibitors. Cell. Signal..

[bib14] Hornak V., Abel R., Okur A., Strockbine B., Roitberg A., Simmerling C. (2006). Comparison of multiple Amber force fields and development of improved protein backbone parameters. Proteins.

[bib15] Ivantsiv Y., Kaplun L., Tzirkin-Goldin R., Shabek N., Raveh D. (2006). Unique role for the UbL-UbA protein Ddi1 in turnover of SCFUfo1 complexes. Mol. Cell Biol..

[bib16] Jorgensen W.L., Chandrasekhar J., Madura J.D., Impey R.W., Klein M.L. (1983). Comparison of simple potential functions for simulating liquid water. J. Chem. Phys..

[bib17] Kaplun L., Ivantsiv Y., Bakhrat A., Tzirkin R., Baranes K., Shabek N., Raveh D. (2006). The F-box protein, Ufo1, maintains genome stability by recruiting the yeast mating switch endonuclease, Ho, for rapid proteasome degradation. Isr. Med. Assoc. J..

[bib18] Koizumi S., Irie T., Hirayama S., Sakurai Y., Yashiroda H., Naguro I., Ichijo H., Hamazaki J., Murata S. (2016). The aspartyl protease DDI2 activates Nrf1 to compensate for proteasome dysfunction. Elife.

[bib19] Kottemann M.C., Conti B.A., Lach F.P., Smogorzewska A. (2018). Removal of RTF2 from stalled replisomes promotes maintenance of genome integrity. Mol. Cell.

[bib20] Krylov D.M., Koonin E.V. (2001). A novel family of predicted retroviral-like aspartyl proteases with a possible key role in eukaryotic cell cycle control. Curr. Biol..

[bib21] Kumar S., Suguna K. (2018). Crystal structure of the retroviral protease-like domain of a protozoal DNA damage-inducible 1 protein. FEBS Open Bio..

[bib22] Lehrbach N.J., Ruvkun G. (2016).

[bib23] Li T.S., Tubiana R., Katlama C., Calvez V., Mohand H.A., Autran B. (1998). Long-lasting recovery in CD4 T-cell function and viral-load reduction after highly active antiretroviral therapy in advanced HIV-1 disease. Lancet.

[bib24] Liu Y., Xiao W. (1997). Bidirectional regulation of two DNA-damage-inducible genes, *MAG1* and *DDI1*, from *Saccharomyces cerevisiae*. Mol. Microbiol..

[bib25] Lustgarten V., Gerst J.E. (1999). Yeast VSM1 encodes a v-SNARE binding protein that may act as a negative regulator of constitutive exocytosis. Mol. Cell Biol..

[bib26] Marash M., Gerst J.E. (2003). Phosphorylation of the autoinhibitory domain of the Sso t-SNAREs promotes binding of the Vsm1 SNARE regulator in yeast. Mol. Biol. Cell.

[bib27] McCoy A.J., Grosse-Kunstleve R.W., Adams P.D., Winn M.D., Storoni L.C., Read R.J. (2007). Phaser crystallographic software. J. Appl. Crystallogr..

[bib28] Morris G.M., Ruth H., Lindstrom W., Sanner M.F., Belew R.K., Goodsell D.S., Olson A.J. (2009). Software news and updates AutoDock4 and AutoDockTools4: automated docking with selective receptor flexibility. J. Comput. Chem..

[bib29] Murshudov G.N., Vagin A.A., Dodson E.J. (1997). Refinement of macromolecular structures by the maximum-likelihood method. Acta Crystallogr. D.

[bib30] Nowicka U., Zhang D., Walker O., Krutauz D., Castañeda C.A., Chaturvedi A., Chen T.Y., Reis N., Glickman M.H., Fushman D. (2015). DNA-damage-inducible 1 protein (Ddi1) contains an uncharacteristic ubiquitin-like domain that binds ubiquitin. Structure.

[bib31] Parrinello M., Rahman A. (1981). Polymorphic transitions in single crystals: a new molecular dynamics method. J. Appl. Phys..

[bib32] Perteguer M.J., Gómez-Puertas P., Cañavate C., Dagger F., Gárate T., Valdivieso E. (2013). Ddi1-like protein from *Leishmania major* is an active aspartyl proteinase. Cell Stress Chaperones.

[bib33] Pettersen E.F., Goddard T.D., Huang C.C., Couch G.S., Greenblatt D.M., Meng E.C., Ferrin T.E. (2004). UCSF Chimera - a visualization system for exploratory research and analysis. J. Comput. Chem..

[bib34] Savoia D., Allice T., Tovo P.A. (2005). Antileishmanial activity of HIV protease inhibitors. Int. J. Antimicrob. Agents.

[bib35] Schymkowitz J., Borg J., Stricher F., Nys R., Rousseau F., Serrano L. (2005). The FoldX web server: an online force field. Nucleic Acids Res..

[bib36] Schmidt W., Wahnschaffe U., Schäfer M., Zippel T., Arvand M., Meyerhans A., Riecken E.O., Ullrich R. (2001).

[bib37] Serbyn N., Noireterre A., Bagdiul I., Plank M., Michel A.H., Loewith R., Kornmann B., Stutz F. (2020). The aspartic protease Ddi1 contributes to DNA-protein crosslink repair in yeast. Mol. Cell.

[bib38] Sievers F., Higgins D.G. (2018). Clustal Omega for making accurate alignments of many protein sequences. Protein Sci..

[bib39] Sirkis R., Gerst J.E., Fass D. (2006). Ddi1, a eukaryotic protein with the retroviral protease fold. J. Mol. Biol..

[bib40] Sivá M., Svoboda M., Veverka V., Trempe J.F., Hofmann K., Kožíšek M., Hexnerová R., Sedlák F., Belza J., Brynda J., Šácha P., Hubálek M., Starková J., Flaisigová I., Konvalinka J., Šašková K.G. (2016). Human DNA-damage-inducible 2 protein is structurally and functionally distinct from its yeast ortholog. Sci. Rep..

[bib41] Suhre K., Sanejouand Y.H. (2004). ElNémo: a normal mode web server for protein movement analysis and the generation of templates for molecular replacement. Nucleic Acids Res..

[bib42] Svoboda M., Konvalinka J., Trempe J.F., Grantz Saskova K. (2019). The yeast proteases Ddi1 and Wss1 are both involved in the DNA replication stress response. DNA Repair.

[bib43] Trempe J.F., Šašková K.G., Sivá M., Ratcliffe C.D.H., Veverka V., Hoegl A., Ménade M., Feng X., Shenker S., Svoboda M., Kozíšek M., Konvalinka J., Gehring K. (2016). Structural studies of the yeast DNA damage-inducible protein Ddi1 reveal domain architecture of this eukaryotic protein family. Sci. Rep..

[bib44] Trudel N., Garg R., Messier N., Sundar S., Ouellette M., Tremblay M.J. (2008). Intracellular survival of Leishmania species that cause visceral Leishmaniasis is significantly reduced by HIV-1 protease inhibitors. J. Infect. Dis..

[bib45] Valdivieso E., Dagger F., Rascón A. (2007). *Leishmania mexicana*: identification and characterization of an aspartyl proteinase activity. Exp. Parasitol..

[bib46] White R.E., Powell D.J., Berry C. (2011). HIV proteinase inhibitors target the Ddi1-like protein of Leishmania parasites. Faseb. J..

[bib47] Winn M.D., Ballard C.C., Cowtan K.D., Dodson E.J., Emsley P., Evans P.R., Keegan R.M., Krissinel E.B., Leslie A.G.W., McCoy A., McNicholas S.J., Murshudov G.N., Pannu N.S., Potterton E.A., Powell H.R., Read R.J., Vagin A., Wilson K.S. (2011). Overview of the CCP4 suite and current developments. Acta Crystallogr. D.

[bib48] Wang J.-L., Elsheikha H.M., Li T.-T., He J.-J., Bai M.-J., Liang Q.-L., Zhu X.-Q., Cong W. (2019). Efficacy of antiretroviral compounds against *Toxoplasma gondii* in vitro. Int. J. Antimicrob. Agents.

[bib49] Yip M.C.J., Bodnar N.O., Rapoport T.A. (2020). Ddi1 is a ubiquitin-dependent protease. Proc. Natl. Acad. Sci. U. S. A.

[bib50] Yu Y., Wang J., Shao Q., Shi J., Zhu W. (2015). Effects of drug-resistant mutations on the dynamic properties of HIV-1 protease and inhibition by Amprenavir and Darunavir. Sci. Rep..

[bib51] Zhang H., Liu J., Ying Z., Li S., Wu Y., Liu Q. (2020). *Toxoplasma gondii* UBL-UBA shuttle proteins contribute to the degradation of ubiquitinylated proteins and are important for synchronous cell division and virulence. Faseb. J..

