# Supplementary material for: Structural and functional insights into the DNA damage-inducible protein 1 (Ddi1) from protozoa
Source: Curr Res Struct Biol. 2022 May 26;4:175–91. doi: 10.1016/j.crstbi.2022.05.003 (PMC9168383; doi:10.1016/j.crstbi.2022.05.003)
Supplement: Multimedia component 1 [file mmc1.docx]

**Supporting Information**

**Structural and functional insights into the DNA damage-inducible protein 1 (Ddi1) from protozoa**

Killivalavan Asaithambi^1^, Iman Biswas^1^, Kaza Suguna^*^

Molecular Biophysics Unit, Indian Institute of Science, Bangalore 560012, India

^*^ Corresponding author at: Molecular Biophysics Unit, Indian Institute of Science, Bangalore 560012, India.

Email: suguna@iisc.ac.in

^1^ Equal contribution


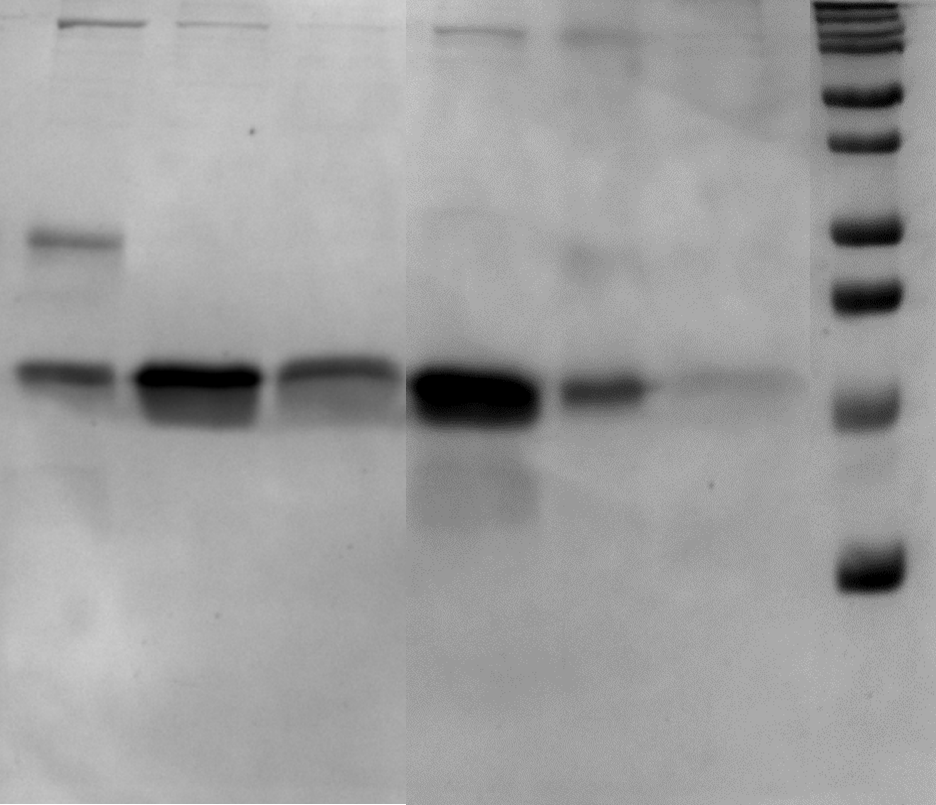


**Toxo_WT**

**Toxo_D315N**

**Toxo_D315A**

**Cryp_WT**

**Cryp_D220N**

**Cryp_D220A**

**M**

**100**

**10**

**15**

**20**

**25**

**37**

**50**

**75**

**150**

**250**

**Supplementary Fig. 1**. SDS-PAGE of *Toxo*Ddi1-RVP, *Cryp*Ddi1-RVP and their mutants; molecular weight corresponding to ~17 kDa. M: Marker. Molecular weight in kDa.


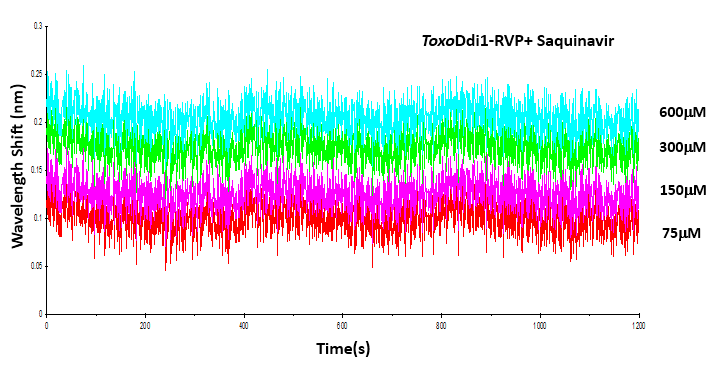


***Toxo*Ddi1-RVP + Saquinavir**


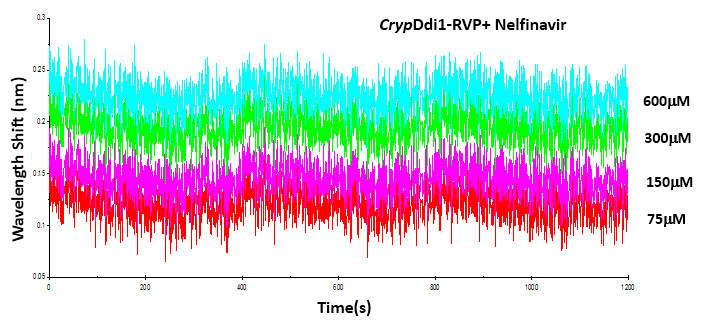


***Cryp*Ddi1-RVP + Nelfinavir**


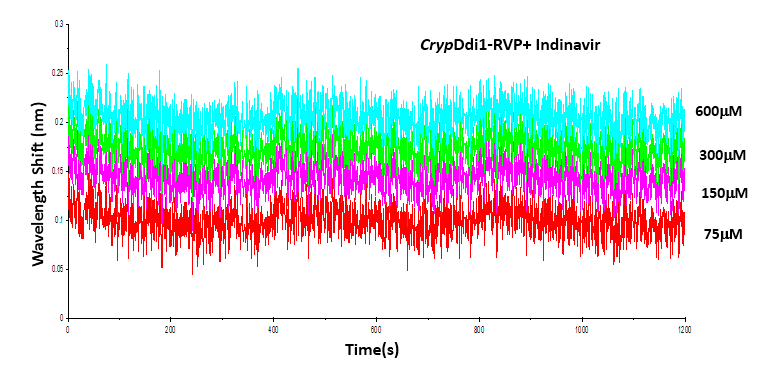

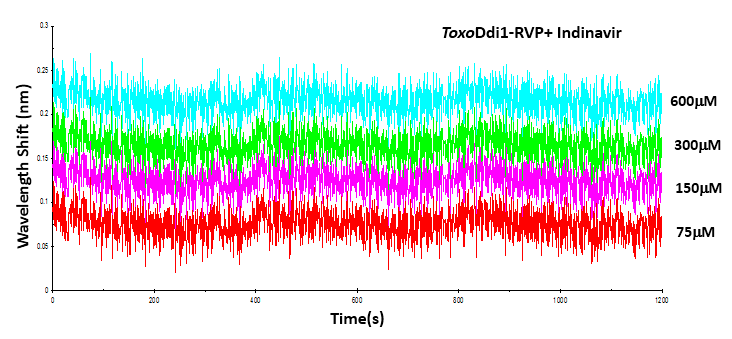


***Cryp*Ddi1-RVP + Indinavir**

***Toxo*Ddi1-RVP + Indinavir**

**Supplementary Fig. 2.** BLI binding profiles of inhibitors that do not bind to the Ddi1-RVP domains.

**
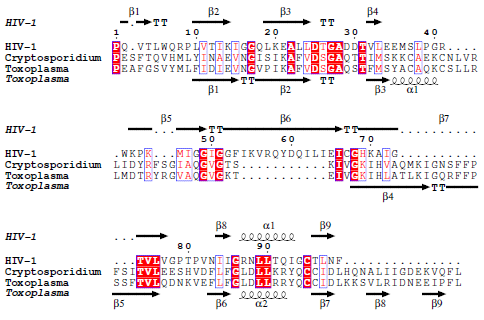
**

**Supplementary Fig. 3.** Structure-based sequence alignment of HIV protease with *Toxo*Ddi1-RVP and *Cryp*Ddi1-RVP.

**Active site interaction in *Toxo*Ddi1-RVP**

**“fireman’s grip” in *Toxo*Ddi1-RVP dimer**


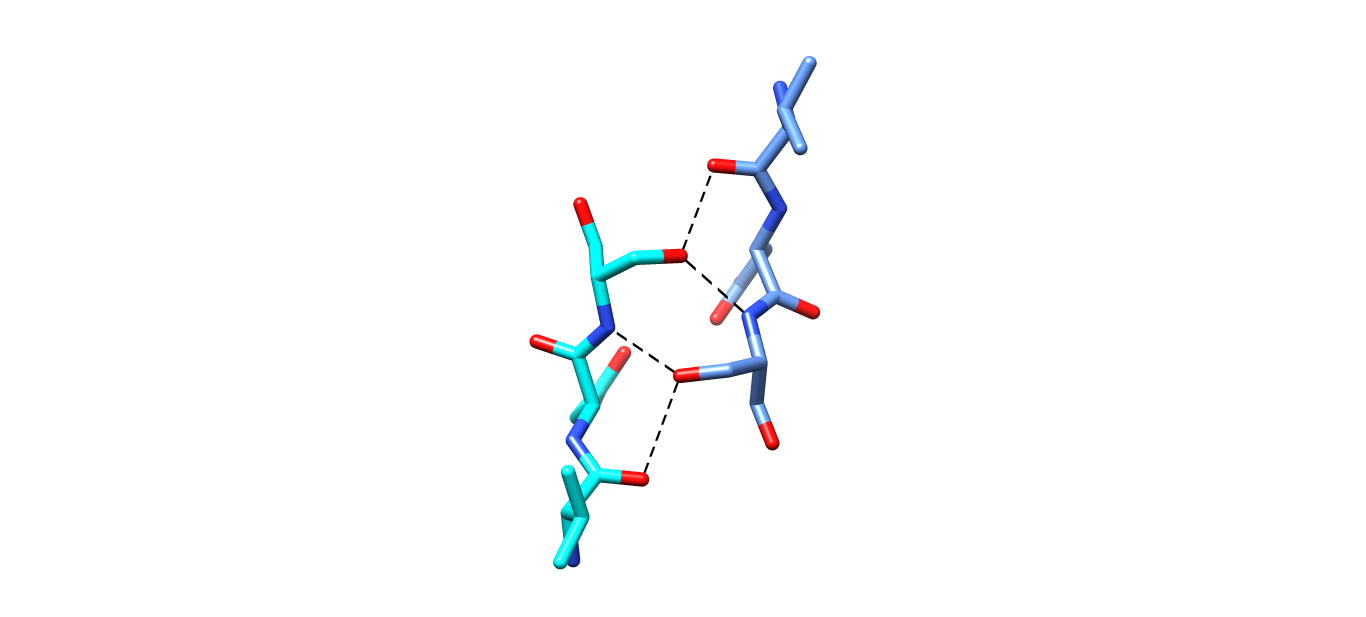


**V314’**

**V314**

**D315**

**D315’**

**S316**

**S316’**


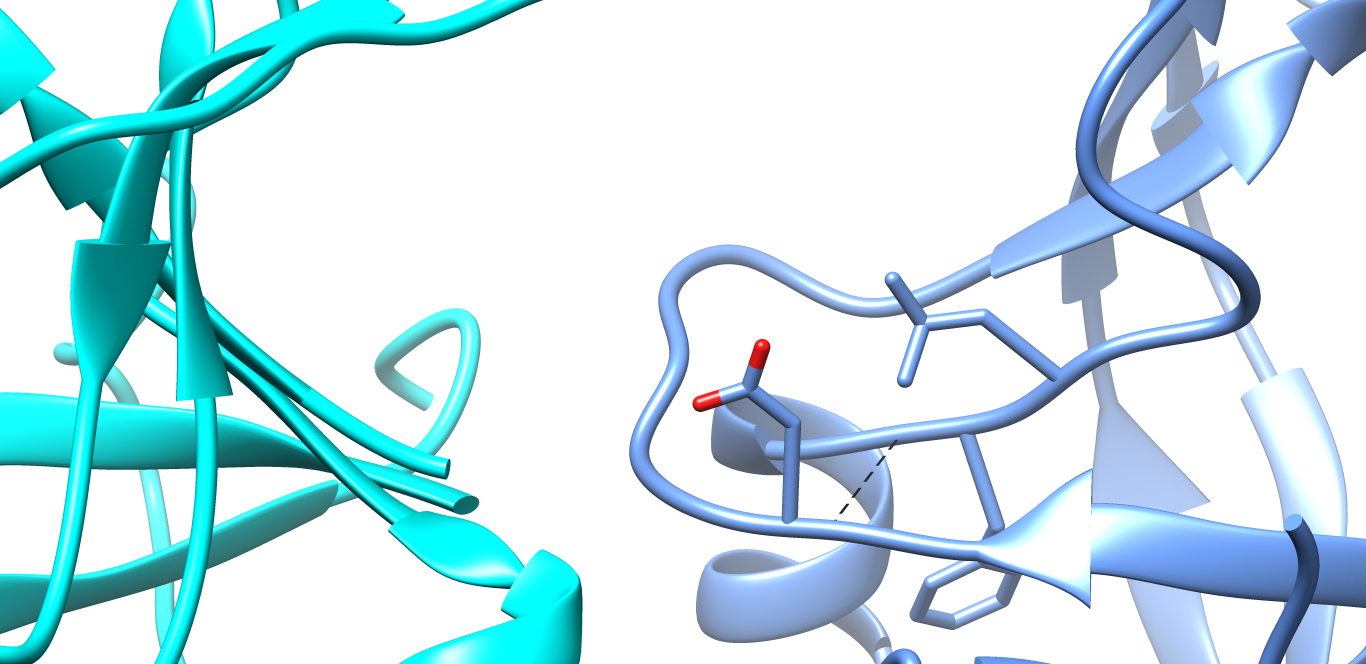


**G386**

**F385**

**L384**

**D315**

**A**

**B**

***Toxo*Ddi1-RVP**

***Cryp*Ddi1-RVP**

**Binding pocket in *Toxo*Ddi1-RVP dimer**


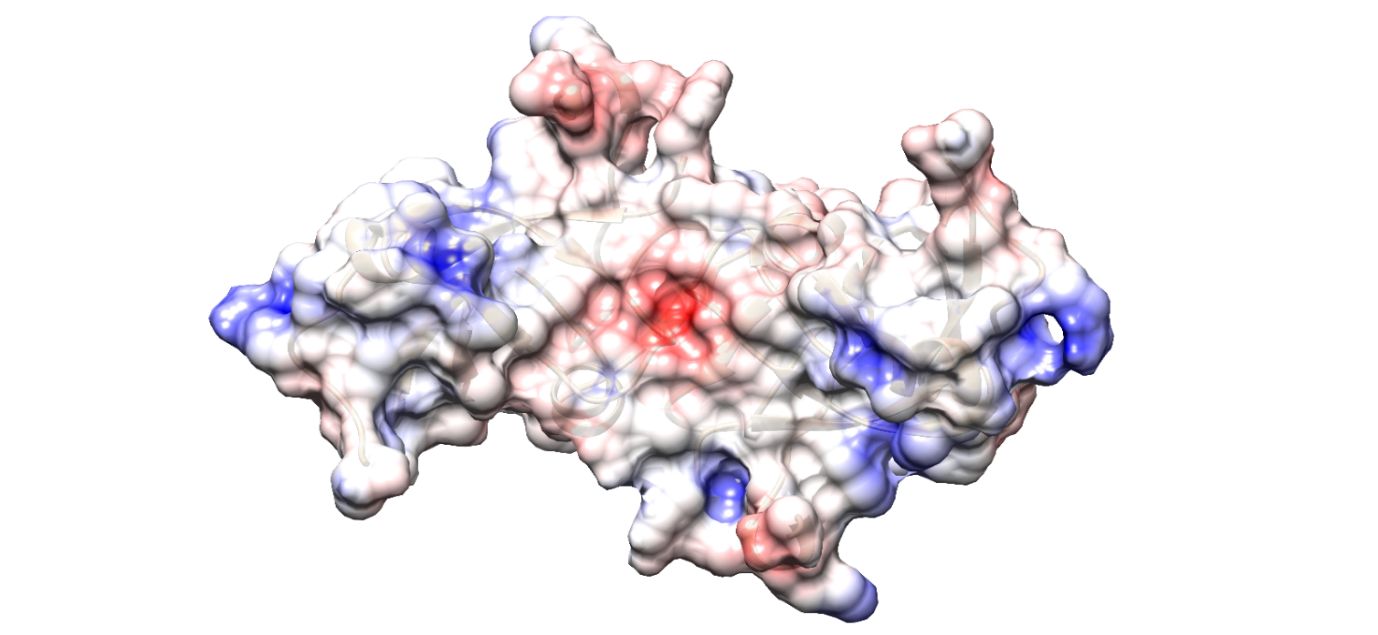


**D315**

**D315**

**F313**

**F300**

**M298**

**A322**

**A376**

**F300**

**M298**

**F313**

**A376**

**A322**


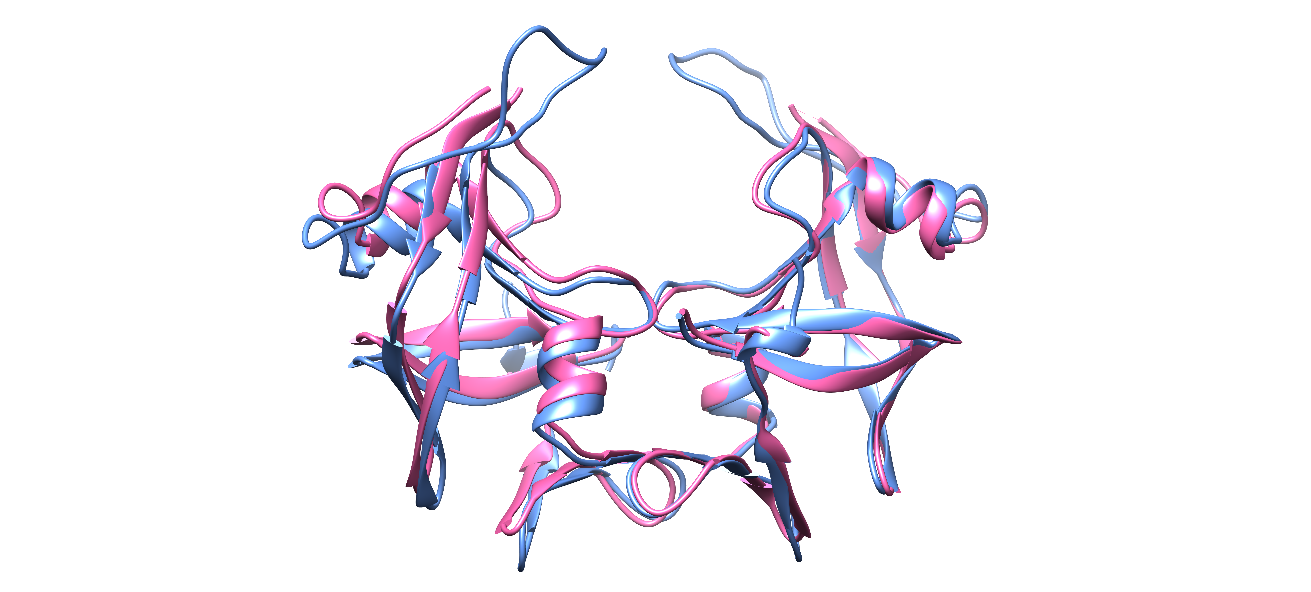


**27 Å**

**22.5 Å**

**C**

**D**

**Supplementary Fig. 4.** Structural details of *Toxo*Ddi1-RVP. **(**A) The “fireman’s grip” arrangement (B) Interaction of the HHG motif with the active site aspartate (C) Surface representation of the binding pocket (D) Superposition of the dimers of *Toxo*Ddi1-RVP (blue) and *Cryp*Ddi1-RVP (pink), highlighting the difference in the size of their binding cavities, measured between Asn379 and Asn379’ of *Toxo*Ddi1-RVP and Ser284 and Ser284’ of *Cryp*Ddi1-RVP.
